# The Discovery of Novel Biomarkers Improves Breast Cancer Intrinsic Subtype Prediction and Reconciles the Labels in the METABRIC Data Set

**DOI:** 10.1371/journal.pone.0129711

**Published:** 2015-07-01

**Authors:** Heloisa Helena Milioli, Renato Vimieiro, Carlos Riveros, Inna Tishchenko, Regina Berretta, Pablo Moscato

**Affiliations:** 1 Priority Research Centre for Bioinformatics, Biomarker Discovery and Information-Based Medicine, Hunter Medical Research Institute, New Lambton Heights, NSW, Australia; 2 School of Environmental and Life Science, The University of Newcastle, Callaghan, NSW, Australia; 3 Centro de Informática, Universidade Federal de Pernambuco, Recife, PE, Brazil; 4 School of Electrical Engineering and Computer Science, The University of Newcastle, Callaghan, NSW, Australia; ENEA, ITALY

## Abstract

**Background:**

The prediction of breast cancer intrinsic subtypes has been introduced as a valuable strategy to determine patient diagnosis and prognosis, and therapy response. The PAM50 method, based on the expression levels of 50 genes, uses a single sample predictor model to assign subtype labels to samples. Intrinsic errors reported within this assay demonstrate the challenge of identifying and understanding the breast cancer groups. In this study, we aim to: a) identify novel biomarkers for subtype individuation by exploring the competence of a newly proposed method named CM1 score, and b) apply an ensemble learning, as opposed to the use of a single classifier, for sample subtype assignment. The overarching objective is to improve class prediction.

**Methods and Findings:**

The microarray transcriptome data sets used in this study are: the METABRIC breast cancer data recorded for over 2000 patients, and the public integrated source from ROCK database with 1570 samples. We first computed the CM1 score to identify the probes with highly discriminative patterns of expression across samples of each intrinsic subtype. We further assessed the ability of 42 selected probes on assigning correct subtype labels using 24 different classifiers from the Weka software suite. For comparison, the same method was applied on the list of 50 genes from the PAM50 method.

**Conclusions:**

The CM1 score portrayed 30 novel biomarkers for predicting breast cancer subtypes, with the confirmation of the role of 12 well-established genes. Intrinsic subtypes assigned using the CM1 list and the ensemble of classifiers are more consistent and homogeneous than the original PAM50 labels. The new subtypes show accurate distributions of current clinical markers ER, PR and HER2, and survival curves in the METABRIC and ROCK data sets. Remarkably, the paradoxical attribution of the original labels reinforces the limitations of employing a single sample classifiers to predict breast cancer intrinsic subtypes.

## Introduction

Breast cancer has been perceived as several distinct diseases characterised by intrinsic aberrations, heterogeneous behaviour and divergent clinical outcome [1]. The classification of breast cancer in discernible molecular subtypes has motivated translational researchers in the past decades towards the design of patient prognosis and the development of tailored treatments [2]. In this scenario, the analysis of breast tumours using microarray data has significantly improved the disease taxonomy and the discovery of new biomarkers for implementation in clinical practice [3–6]. In the early 2000s, five intrinsic subtypes were proposed: *luminal A, luminal B, HER2-enriched, normal-like* and *basal-like* breast tumours [7, 8]. Following this initial molecular taxonomy, further sub-classifications of breast cancer in distinct entities have been suggested [9–11].

The transcriptomic patterns observed across subtypes has given us insight into the molecular complexity and inherent alterations in tumour cells modelling the breast cancer heterogeneity and unpredicted outcome [12, 13]. Strikingly, intrinsic gene lists have been explored to reliably assign breast tumour samples into formal molecular subtypes, survival rate and treatment outline [3, 7, 8, 14–18]. Recently, Parker and colleagues [16] proposed a list of 50 genes that together with the Prediction Analysis for Microarrays (PAM) classification algorithm [19] aimed at identifying subtypes and enlarging the prognostic information with high potential for validation in clinical settings [16, 20, 21]. The resulting technique, called the PAM50 method, has been widely applied to categorize tumours into one of the five classical intrinsic subtypes.

Although independent cohorts attempted to identify molecular subtypes, the chosen microarray-based Single Sample Predictor (SSP) model revealed unreliable assignments and modest agreement between studies [21, 22]. In fact, the perceived inability of some analytical methods to deal with the challenges of processing high-dimensional data, in addition to the difficulties on validating independent/unpaired technologies may limit the precise characterisation of the subtypes [21, 23, 24]. Therefore, novel methods are urgently needed in order to provide better tumour stratification and accurate biomarkers identification [25, 26]. In this scenario, the high quality of the microarray gene expression data set processed by the Molecular Taxonomy of Breast Cancer International Consortium (METABRIC) [27], with over 2000 samples, offers a unique opportunity to refine and expand the list of transcripts that best discriminate intrinsic subtypes. A precise classification of breast tumours, consequently, would lead to improvements in the valuation of the disease, currently guided by oestrogen and progesterone receptor (ER and PR) status, and HER2 amplification [24, 28].

In this report, we focus on the use of a ranking feature method based on the newly proposed CM1 score [29] to identify probe sets that appear naturally from the METABRIC breast cancer data set. For doing so, we use the entire set of 48803 probes as an alternative to the selection from pre-existing literature as performed by other authors [15, 16]. Moreover, the quality of the probes for predicting subtypes is carefully appraised in the METABRIC data set (Illumina BeadArray) and further validated in different studies (Affymetrix GeneChip) accessed through the Research Online Cancer Knowledgebase (ROCK) interface [30]. However, instead of relying on a single method to assign sample subtype, as suggested by Parker et al. (2009) [16] with the PAM50 method, we explore an ensemble learning. Our analysis is based on the performance of a large set of classification models from the Weka software suite [31]; a technique previously recommended by Ravetti and Moscato [32]. The classifiers are used in combination with the list of probes selected using CM1 score and, alternatively, with the 50 genes from the PAM50 commercial assay [16]. We also compute several statistical measures to determine the power of both lists on predicting breast cancer subtypes. Ultimately, we correlate the study outcomes within current clinical information and survival analysis.

## Materials and Methods

### Data sets description

The METABRIC microarray data set used in this study is hosted by the European Bioinformatics Institute (EBI) and deposited in the European Genome-Phenome Archive (EGA) at http://www.ebi.ac.uk/ega/, under accession number EGAS00000000083. It consists of transcriptomic information (cDNA microarrays profiling) processed on the Illumina HT-12 v3 platform (Illumina_Human_WG-v3), as described in [27]. The log_2_-normalised gene expression values of primary tumours were divided into two subsets by METABRIC: *discovery* (997 samples) and *validation* (989 samples), which were respectively used as *training* and *test* sets in our experiments. The original study collected and analysed data under the approval of the ethics Institutional Review Board (details in [27]). The use of this data for research was also approved by the Human Ethics Research Committee (HREC) of The University of Newcastle, Australia, (approval number: H-2013–0277).

The second data set is publicly available in ROCK online portal [30] at http://rock.icr.ac.uk/, under data source access GSE47561. This source integrates ten data studies (GSE2034, GSE11121, GSE20194, GSE1456, GSE2603, GSE6532, GSE20437, E-TABM-185, GSE7390, GSE5847) performed on the Affymetrix Human Genome U133A Array (HG-U133A) platform. The matrix contains log2 RMA re-normalised gene expression data in a unique comprehensive report of 1570 samples. Thus, the GSE47561 data set was used as a second validation set to test our method.

In brief, both METABRIC and ROCK data sets have information on patients’ long-term clinical and pathological outcomes, including the sample assignment into intrinsic subtypes (luminal A, luminal B, HER2-enriched, normal-like, and basal-like) according to the PAM50 method [16]. The METABRIC data set has a more comprehensive description of patient clinical features, whereas the ROCK data set presents no standardized information across the ten different studies.

### Study Design and Computing Resources

**Fig 1:**
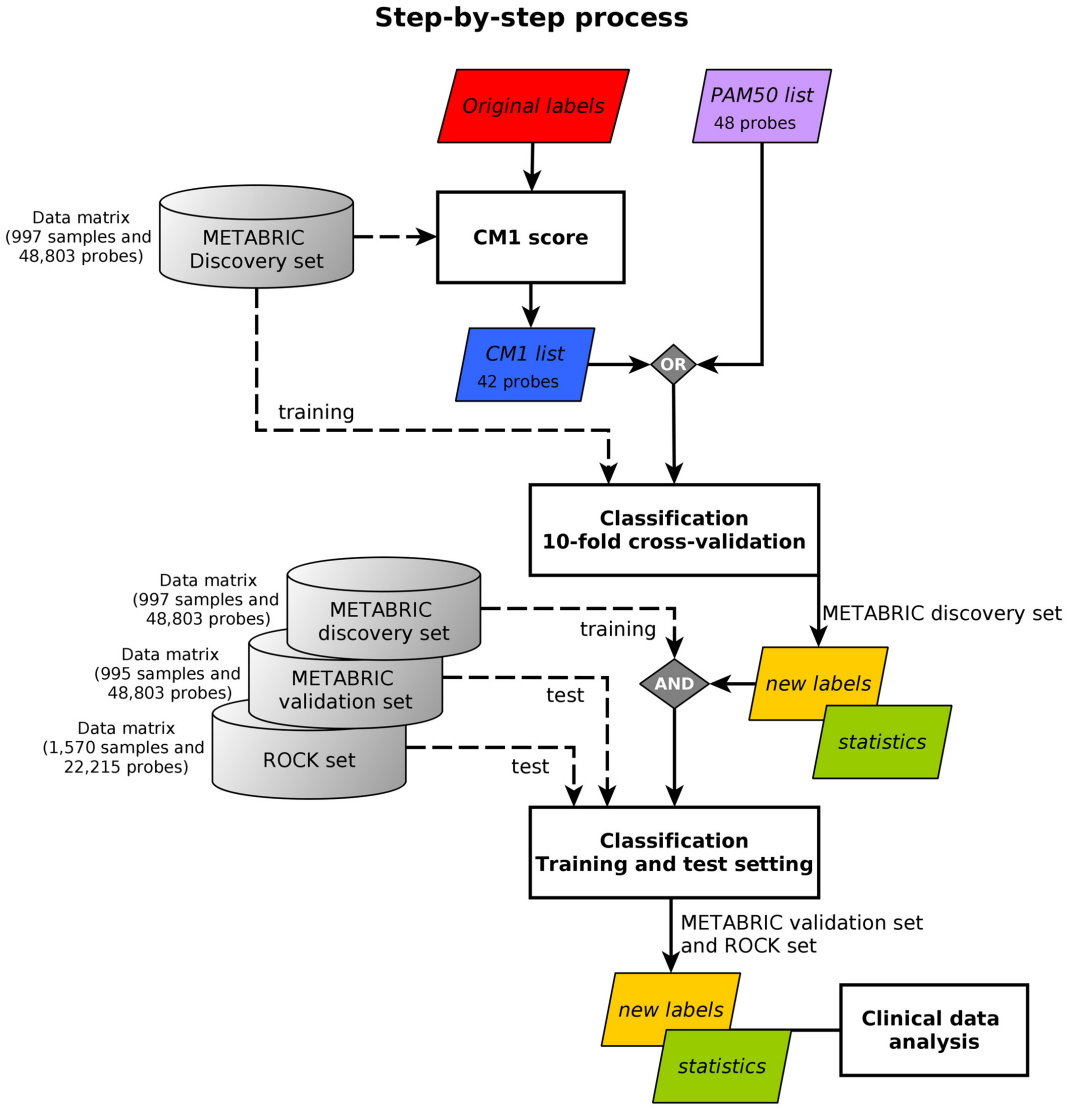
The step-by-step process. The image shows the method steps based on *CM1 score* and *ensemble learning*. The *METABRIC discovery set* is used to compute the *CM1 score*, based on the *original labels* previously assigned with the PAM50 method. This step has an output of 42 discriminative probes selected, the *CM1 list*. The following step involve the sample subtype classification based on a *10-fold cross-validation*. Samples in the METABRIC discovery set are considered to train 24 classifiers using the CM1 list and, alternatively, the *PAM50 list*. The samples are partitioned into ten folds; then a model is built using 90% of samples, which is used to predict the labels of the remaining 10%. After the ten turns are finished, the level of association between the predicted and original METABRIC labels is computed using several *statistics*. In the *training-test setting*, labels of samples in the *METABRIC validation set* and *ROCK set* are predicted with the models built in the discovery. Statistics measurements are again computed to assess the model performance on predicting breast cancer subtypes. In both classification steps, the new labels are attributed based on the consensus of the majority of the classifiers. Finally, the results or new labels are compared against the *clinical data*, the current markers ER, PR and HER2, and survival curves.

In this study, we propose a systematic approach that aims at improving breast cancer subtype prediction. The systematic approach is built based on feature selection and data mining concepts. We first compute the CM1 score—using the microarray mRNA expression values—to rank the whole set of probes based on their discriminative power across breast cancer subtypes. We then select the top 10 probes that best represent each intrinsic subtype. The quality of this selection is assessed using a set of classifiers from the Weka software suite with the METABRIC and ROCK data sets, followed by the statistical analysis. The process flow is depicted in Fig 1, and further explained in the remainder of this section.

### Selection of biomarkers using the CM1 score

The CM1 score is a supervised univariate method used to measure the difference in expression levels of samples in two different classes [29]. In this study, it is used as a ranking feature to select a subset of highly discriminative probes for each breast cancer intrinsic subtype. Let *X* and *Y* be a partition of a set of samples into two classes, with *X* the *class of interest* and *Y* the *remaining classes*. A sample either belongs to *X* or to *Y*. For each probe *i* we compute the CM1 score as:

$$\begin{array}{c} CM1_{i}\left(X,Y\right)=\frac{{\bar{x}}_{i}-{\bar{y}}_{i}}{1+(max\left\{y_{i}\right\}-min\left\{y_{i}\right\})} \end{array}$$ (1)

where ${\bar{x}}_{i}$ is the average expression value of the probe *i* for samples in class *X*, ${\bar{y}}_{i}$ is the average expression value of the probe *i* for samples in class *Y*; *max*{*y*_*i*_} and *min*{*y*_*i*_} are the minimum and maximum expression values of the probe *i* for samples in the class *Y*. Eq 1 can be interpreted as the normalised difference between the averages of expression values in the class *X* and *Y*. The normalisation is proportional to the range of values in *Y*.

To define the most discriminative probes for each breast cancer subtype (luminal A, luminal B, HER2-enriched, normal-like and basal-like), we computed the CM1 score for each of 48803 probes taking the subtype of interest and the remaining ones. This results in 5 lists of 48803 CM1 scores.

Considering the fact that Parker et al. (2009) [16] were able to define the five breast cancer classes based on 50 genes, for each subtype we chose the 10 most important probes (5 with the greatest positive CM1 score values—indicating up-regulated probes relative to the other subtypes –, and 5 with the smallest negative values – representing down-regulation). This set is referred to as the *balanced top ten* in this paper. Collecting the balanced top ten lists of all subtypes leads to a new set of 42 unique Illumina probes, meaning that 8 probes appear in multiple subtypes. This list is hereafter called the *CM1 list*.

### Assessment of the quality of the CM1 list based on ensemble learning

The quality of the CM1 list for distinguishing subtypes was assessed using a list of well-known classifiers available in the Weka data mining software suite [31]. It uses different types of classifiers such as bayesian, functions, lazy, meta, rule-based and decision trees. Each classifier was trained with a subset of the data comprising all samples in the METABRIC discovery set and the 42 probes in the CM1 list using both 10-fold cross-validation and training-test setting. In the 10-fold cross-validation, the samples are first partitioned into ten folds; then a model is built using 90% of samples, which is thereafter used to predict the labels of the remaining 10%. After the ten turns are finished, the level of association between the predicted and original METABRIC labels is computed using Cramer’s V [33]. In the training-test setting, labels of samples in the METABRIC validation set and ROCK data are predicted using models built with the samples in the *discovery set*. The new labels were attributed based on the consensus of the majority of the classifiers (i.e. more than 50% percent), and whenever such condition was not achieved samples were marked as inconsistent (INC).

A similar approach was performed with the PAM50 list to serve as baseline for comparing the results obtained with the 42 probes from the CM1 list. The 50 genes identified by Parker et al. (2009) [16] were mapped to Illumina probes by Curtis et al. (2012) [27], following strict criteria. Only genes and corresponding probe with perfect annotation [34] on the Illumina HT-12 v3 BeadChip were considered. Probes containing SNPs, multiple targets or mismatches, or lying in repeat-masked regions were discarded. Finally, a total of 48 probes corresponding to genes in the PAM50 list were selected to conduct the classification experiments as described for the CM1 list. For Affymetrics HG-U133A, the CM1 and PAM50 lists were mapped according to ‘genefu’ R package, using Entrez Gene ID as reference. For instance, the 42 probes from the CM1 list matched 33 probes, whereas the 48 from PAM50 list paired 43 probes in the Affymetrix platform. In case of multiple mappings the probe with the most variation was selected according to the ‘genefu’ instructions. Before testing the classifiers in ROCK data set, the Affymetrix and Illumina expression levels were min-max normalised.

### The statistical analysis

#### Cramer’s V

Given a *r* × *c* contingency table describing the association between the original labels and those predicted by the majority of classifiers, Cramer’s V measures the level of association between those two nominal variables. The statistic ranges from 0, representing no association between the two variables, to 1, representing complete association. Cramer’s V is computed using Eq 2.

$$\begin{array}{c} \phi =\sqrt{\frac{\chi^{2}}{N\;min\{r-1,c-1\}}} \end{array}$$ (2)

where *N* is the number of samples in the data set, and *χ* ^2^ is Pearson’s chi-squared value.

#### Average sensitivity (AS)

The average sensitivity (AS) [31] was also computed to assess the performance of classifiers with both lists. The AS is the average proportion of accurately classified samples of each subtype. Considering a *r* × *c* contingency table associating initial and predicted labels, the average sensitivity of a classifier is given by Eq 3.

$$\begin{array}{c} AS=\frac{1}{r}\sum \frac{n_{ii}}{n_{i•}} \end{array}$$ (3)

where *r* is the number of classes (subtypes), *n*_*ii*_ is the number of samples of class *i* correctly predicted as *i*, and *n*_*i*•_ is the number of samples of class *i* (row marginals).

#### Fleiss’ kappa

The consensus of the different classification methods concerning the samples’ labels was measured by the popular interrater reliability metric Fleiss’ kappa [35, 36]. The statistic was used to gauge not only the agreement among classifiers trained with the different probe sets, but also between the labels assigned by the majority of classifiers and the original METABRIC labels. It also quantifies the agreement between predicted labels using the CM1 and PAM50 lists.

Assuming a *s* × *c* contingency table informing how many times each of the *c* classes were assigned to each of the *s* samples in the *k* different sample labellings, the Fleiss’ kappa statistic is computed as defined by Eq 4.

$$\begin{array}{c} \kappa =\frac{\sum \sum n_{ij}^{2}-sk[1+\left(k-1\right)\sum p_{j}^{2}]}{sk\left(k-1\right)\left(1-\sum p_{j}^{2}\right)} \end{array}$$ (4)

where *n*_*ij*_ contains the number of times sample *i* was assigned label *j*, ∑_*j*_ *n*_*ij*_ = *k*, and *p*_*j*_ = (∑_*i*_ *n*_*ij*_)/*sk* is the probability with which the label *j* is assigned to a sample.

Kappa values range from $\left[-\sum p_{j}^{2}/(1-\sum p_{j}^{2})\right]$ to +1, which, according to Landis and Koch’s division [37], can be interpreted in the following manner: (1) values below zero are considered *poor agreement*; (2) values between zero and 0.2 are considered *slight agreement*; (3) 0.21 ≤ *κ* ≤ 0.40 is *fair agreement*; (4) 0.41 ≤ *κ* ≤ 0.60 *moderate agreement*; (5) 0.61 ≤ *κ* ≤ 0.80 *substantial agreement*; and (6) 0.81 ≤ *κ* ≤ 1 is regarded as an *almost perfect agreement*.

#### Adjusted Rand Index

The agreement between pairs of sample labellings was also quantified using this metric. It ranges between 0 to 1, where 1 indicates an almost perfect concordance between the two compared bipartitions, and 0 a complete discordance between them. The *Adjusted Rand Index* is a version of Rand index corrected for chance when the partitions are picked at random [38, 39]. Given a *r* × *c* contingency table between two labelling *R* and *C*, it can be measured by:

ARI(R,C)=∑ij(nij2)-[∑i(ni•2)∑j(n•j2)]/(N2)12[∑i(ni•2)+∑j(n•j2)]-[∑i(ni•2)∑j(n•j2)]/(N2) (5)

where 1 ≤ *i* ≤ *r*, 1 ≤ *j* ≤ *c*, and *n*_*ij*_ is an entry of the contingency table representing the number of samples that are in class *R*_*i*_ in the partition *R* and *C*_*j*_ in the partition *C*, *n*_*i*•_ and *n*_•*j*_ are the table’s marginals.

### Survival analysis

The survival analysis for each breast cancer subtype is performed using Cox proportional hazards model from the package *survival* in the R software [40, 41]. Only patients who either died due to the disease or are still alive are considered for model estimation. The clinical parameters relevant for the survival study are chosen in correspondence with Curtis et al. (2012) [27]: age at the time of diagnosis, tumor size, tumor grade, the number of positive lymph nodes and ER status according to immunohistochemistry. Since the probability model based on the observations available at certain time points becomes less and less reliable with the increasing time, the median survival lines based on the last 10 observations are plotted in dash. Due to the compilation of ten different studies and the existence of significant gaps in patients’ clinical information, the survival curves in the ROCK data set are not representative across subtypes. In particular, the number of patients with information about *overall survival* and *disease free survival* is limited to only 405, with no specification on the cause of death (i.e. if due to disease or not).

## Results

### Section description and resources

To understand the results described in this section, we introduce the sequence of our approach which combines the *CM1 score* and *ensemble learning*. First, we detail the selection of discriminative probes ranked according to the CM1 score; calculated for each of the five breast cancer subtypes. Second, we show the quality of our probes by using 24 classification models based on a 10-fold cross-validation and training-test setting in the METABRIC and ROCK data sets. The same approach is also performed with the list of 50 genes used in the PAM50 method. In addition, statistical analysis are reported to determine the power of both lists on predicting breast cancer subtypes. Finally, we demonstrate the consistency between the new labels assigned with current clinical markers ER, PR and *HER2*, and survival curves. The step-by-step approach is detailed in the Materials and Methods section.

### Using the CM1 list to differentiate the five intrinsic breast cancer subtypes

The CM1 score was applied to rank the set of 48803 probes for each of the five subtypes in the METABRIC discovery data set (Supporting Information S1 Table). It is important to remark that this method used the original PAM50 subtypes attributed to samples in the METABRIC discovery set. The purpose of doing so is to provide a better molecular characterisation of each class using the wealth of the METABRIC transcriptomic data, besides improving the breast cancer subtype prediction. The probes with the top five negative and top five positive CM1 scores were selected for each subtype. Here, we aimed at obtaining 50 probes that appear naturally from a rich and unique data set. We would then be able to compare such a list with the list of 50 genes embedded in the PAM50 method [16]—the PAM50 list. The final list comprising the union of the top ranked probes is displayed in Table 1, and their CM1 scores and ranks in each subtype in Table 2. Some of the 50 probes selected, however, discriminate more than one subtype and resulted in a list of 42 unique elements, the *CM1 list*. Our selection includes 30 novel biomarkers, while the remaining 12 genes are common with the PAM50 list.

**Table 1:** CM1 list.

Probe ID	Gene name	Gene symbol and aliases	[Refs.]
ILMN_1684217	Aurora kinase B	**AURKB**; AIK2, AIM1, ARK2, AurB, IPL1, STK5, AIM-1, STK12, PPP1R48, aurkb-sv1, aurkb-sv2	[42–54]
ILMN_1683450	Cell division cycle associated 5	**CDCA5**; SORORIN	[55–58]
ILMN_1747016	Centrosomal protein 55kDa	**CEP55**; CT111, URCC6, C10orf3	[59–62]
ILMN_2212909	Maternal embryonic leucine zipper kinase	**MELK**; HPK38	[63–69]
ILMN_1714730	Ubiquitin-conjugating enzyme E2C	**UBE2C**; UBCH10, dJ447F3.2	[70–74]
ILMN_1796059	Ankyrin repeat domain 30A	**ANKRD30A**; NY-BR-1, RP11–20F24.1	[75–82]
ILMN_1651329	Long intergenic non-protein coding RNA 993	**LINC00993**	
ILMN_2310814	Microtubule-associated protein tau	**MAPT**; TAU, MSTD, PPND, DDPAC, MAPTL, MTBT1, MTBT2, FTDP-17	[83–89]
ILMN_1728787	Anterior gradient 3	**AGR3**; HAG3, hAG-3, BCMP11, PDIA18	[90–92]
ILMN_1688071	N-acetyltransferase 1	**NAT1**; AAC1, MNAT, NATI, NAT-1	[93–95]
ILMN_1729216	Crystallin, alpha B	**CRYAB**; MFM2, CRYA2, CTPP2, HSPB5, CMD1II, CTRCT16	[96–99]
ILMN_1666845	Keratin 17	**KRT17**; PC, K17, PC2, PCHC1	[100, 101]
ILMN_1786720	Prominin 1	**PROM1**; RP41, AC133, CD133, MCDR2, STGD4, CORD12, PROML1, MSTP061	[102–106]
ILMN_1753101	V-set domain containing T cell activation inhibitor 1	**VTCN1**; B7X, B7H4, B7S1, B7–H4, B7h.5, VCTN1, PRO1291, RP11–229A19.4	[107–111]
ILMN_1798108	Chromosome 6 open reading frame 211	**C6orf211**	
ILMN_1747911	Cyclin-dependent kinase 1	**CDK1**; CDC2, CDC28A, P34CDC2	[112–116]
ILMN_1666305	Cyclin-dependent kinase inhibitor 3	**CDKN3**; KAP, CDI1, CIP2, KAP1	[117]
ILMN_1678535	Estrogen receptor 1	**ESR1**; ER, ESR, Era, ESRA, ESTRR, NR3A1	[118–121]
ILMN_2149164	Secreted frizzled-related protein 1	**SFRP1**; FRP, FRP1, FrzA, FRP-1, SARP2	[122–139]
ILMN_1788874	Serpin peptidase inhibitor, clade A (alpha-1 antiproteinase, antitrypsin), member 3	**SERPINA3**; ACT, AACT, GIG24, GIG25	[140–143]
ILMN_1785570	Sushi domain containing 3	**SUSD3**	[141, 144]
ILMN_1803236	Chloride channel accessory 2	**CLCA2**; CACC, CACC3, CLCRG2, CaCC-3	[145–148]
ILMN_2161820	Glycine-N-acyltransferase-like 2	**GLYATL2**; GATF-B, BXMAS2–10	[149, 150]
ILMN_1810978	Mucin-like 1	**MUCL1**; SBEM	[151–156]
ILMN_1773459	SRY (sex determining region Y)-box 11	**SOX11**	[157, 158]
ILMN_1674533	Transient receptor potential cation channel, subfamily V, member 6	**TRPV6**; CAT1, CATL, ZFAB, ECAC2, ABP/ZF, LP6728, HSA277909	[159–164]
ILMN_1687235 ILMN_2358760	Hepsin	**HPN**; TMPRSS1	[165]
ILMN_1655915	Matrix metallopeptidase 11 (stromelysin 3)	**MMP11**; ST3, SL-3, STMY3	[166–176]
ILMN_1711470	Ubiquitin-conjugating enzyme E2T (putative)	**UBE2T**; PIG50, HSPC150	[177]
ILMN_1740609	Chemokine (C-C motif) ligand 15	**CCL15**; LKN1, NCC3, SY15, HCC-2, LKN-1, MIP-5, NCC-3, SCYL3, MIP-1D, MRP-2B, SCYA15, HMRP-2B, MIP-1 delta	[178, 179]
ILMN_1789507	Collagen, type XI, alpha 1	**COL11A1**; STL2, COLL6, CO11A1	[180, 181]
ILMN_1651282	Collagen, type XVII, alpha 1	**COL17A1**; BP180, BPA-2, BPAG2, LAD-1, BA16H23.2	[182]
ILMN_1723684	Duffy blood group, atypical chemokine receptor	**DARC**; FY, Dfy, GPD, GpFy, ACKR1, CCBP1, CD234, WBCQ1	[183–186]
ILMN_1809099	Interleukin 33	**IL33**; DVS27, IL1F11, NF-HEV, NFEHEV, C9orf26, RP11–575C20.2	[187]
ILMN_1766650	Forkhead box A1	**FOXA1**; HNF3A, TCF3A	[188–203]
ILMN_1811387	Trefoil factor 3 (intestinal)	**TFF3**; ITF, P1B, TFI	[204–209]
ILMN_1738401	Forkhead box C1	**FOXC1**; ARA, IGDA, IHG1, FKHL7, IRID1, RIEG3, FREAC3, FREAC-3	[210–212]
ILMN_1689146	Gamma-aminobutyric acid (GABA) A receptor, pi	**GABRP**	[213, 214]
ILMN_1807423	Insulin-like growth factor 2 mRNA binding protein 3	**IGF2BP3**; CT98, IMP3, KOC1, IMP-3, VICKZ3	[215–221]
ILMN_1692938	Phosphoserine aminotransferase 1	**PSAT1**; PSA, EPIP, PSAT	[222, 223]
ILMN_1668766	Rhophilin associated tail protein 1	**ROPN1**; CT91, ODF6, ROPN1A, RHPNAP1, ropporin	[224]

**Table 2:** Scores and ranks for the CM1 list.

	Luminal A		Luminal B		Her2		Normal		Basal			
Probe ID	score	rank	score	rank	score	rank	score	rank	score	rank	Symbol	PAM50
ILMN_1728787	0.203	5	0.144	5	-0.314	2		54	-0.461	3	AGR3	
ILMN_1796059	0.216	3		8730		1434		3666	-0.390	5	ANKRD30A	
ILMN_1684217	-0.203	1		74		497		146		97	AURKB	
ILMN_1798108		1980	0.155	2		68		405		179	C6orf211	
ILMN_1740609		476		43		970	0.252	3		2776	CCL15	
ILMN_1747911		80	0.144	4		2080		194		1496	CDC2	
ILMN_1683450	-0.196	3		30		306		79		166	CDCA5	
ILMN_1666305		16	0.146	3		438		167		917	CDKN3	
ILMN_1747016	-0.195	5		88		362		73		127	CEP55	x
ILMN_1803236		1875		354	0.316	3		688		13483	CLCA2	
ILMN_1789507		12176		5363		1820	-0.155	3		9245	COL11A1	
ILMN_1651282		915		16		4821	0.244	4		12205	COL17A1	
ILMN_1729216		6657	-0.153	5		3008		52		45	CRYAB	
ILMN_1723684		456		14		2830	0.255	2		4215	DARC	
ILMN_1678535		8	0.181	1	-0.360	1		7	-0.440	4	ESR1	x
ILMN_1766650		70		85		12522		216	-0.478	2	FOXA1	x
ILMN_1738401		1047		10		2254		226	0.443	1	FOXC1	x
ILMN_1689146		1177		13		1833		283	0.414	2	GABRP	
ILMN_2161820		310		270	0.333	1		791		1479	GLYATL2	
ILMN_1687235		79		1942		58	-0.157	2		211	HPN	
ILMN_2358760		105		1941		73	-0.152	4		284	HPN	
ILMN_1807423		1269		2087		21820		11567	0.405	3	IGF2BP3	
ILMN_1809099		3400		141		6282	0.275	1		23413	IL33	
ILMN_1666845		8365	-0.186	2		3879		35		29	KRT17	x
ILMN_1651329	0.221	1		2481		1149		1159		20	LOC646360	
ILMN_2310814	0.221	2		8776		33		1131		23	MAPT	x
ILMN_2212909	-0.196	4		137		501		92		65	MELK	x
ILMN_1655915		5274		3486		3832	-0.166	1		4148	MMP11	x
ILMN_1810978		20520		9	0.326	2		6		1495	MUCL1	
ILMN_1688071	0.215	4		902	-0.256	5		24		19	NAT1	x
ILMN_1786720		988	-0.174	3		273		465		20	PROM1	
ILMN_1692938		68		343		93		1864	0.391	5	PSAT1	
ILMN_1668766		721		62		1415		368	0.405	4	ROPN1	
ILMN_1788874		148		4633	-0.259	4		1961		1462	SERPINA3	
ILMN_2149164		11497	-0.203	1		1697	0.244	5		40	SFRP1	x
ILMN_1773459		185		621	0.293	5		10046		483	SOX11	
ILMN_1785570		11		2499	-0.308	3		438		82	SUSD3	
ILMN_1811387		26		64		1263		661	-0.521	1	TFF3	
ILMN_1674533		643		605	0.300	4		2756		1819	TRPV6	
ILMN_1714730	-0.200	2		9		318		43		353	UBE2C	x
ILMN_1711470		56		7		1732	-0.145	5		1113	UBE2T	x
ILMN_1753101		474	-0.153	4		2424		3373		1522	VTCN1	

The CM1 scores for the topmost 5 positive and negative probe IDs in each subtype are given. The ranks correspond to the position of the probe from the topmost positive or negative (with 1 being the top ranked score at either side). The rightmost two columns indicate the gene symbol the probe maps to, and which genes appear also in the PAM50 list.

**Fig 2:**
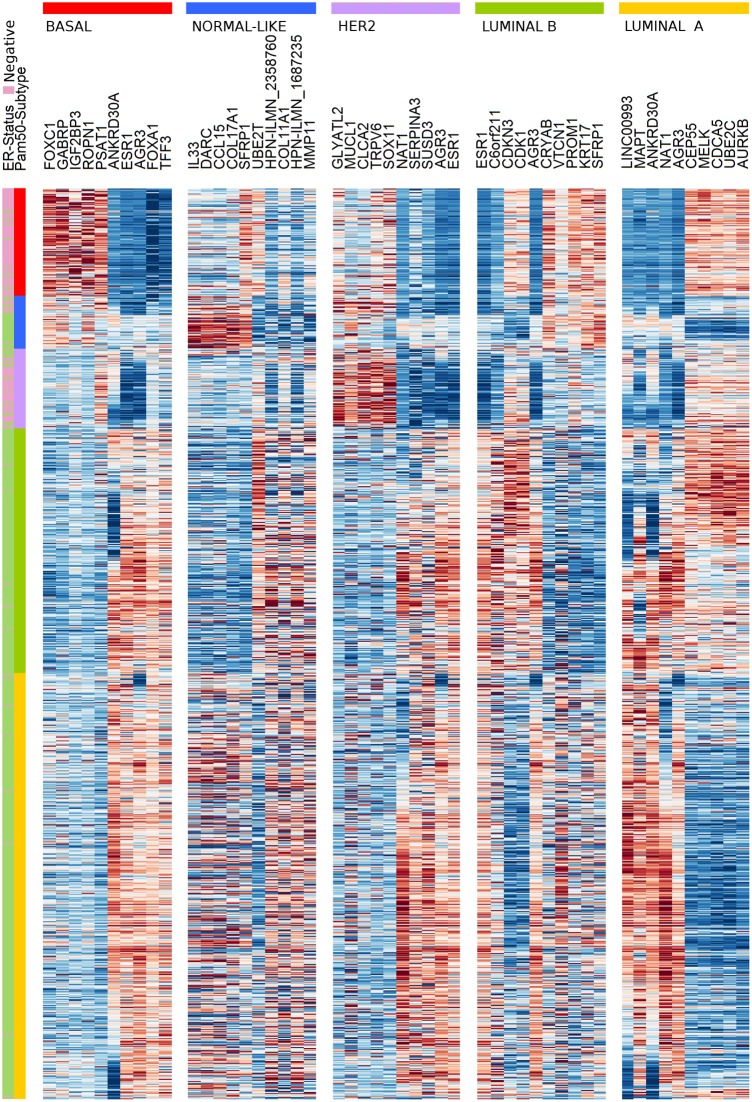
The gene expression profile of the *balanced top ten* probes selected for each of the five breast cancer intrinsic subtypes across 997 samples from the discovery set. The annotated genes are defined for each subtype as an intrinsic, highly discriminative, signature. Samples were ordered according to the gene expression similarities in each breast cancer subtype. Colours represent the selected genes and sample subtypes: luminal A (yellow), luminal B (green), HER2-enriched (purple), normal-like (blue), and basal-like (red).

**Fig 3:**
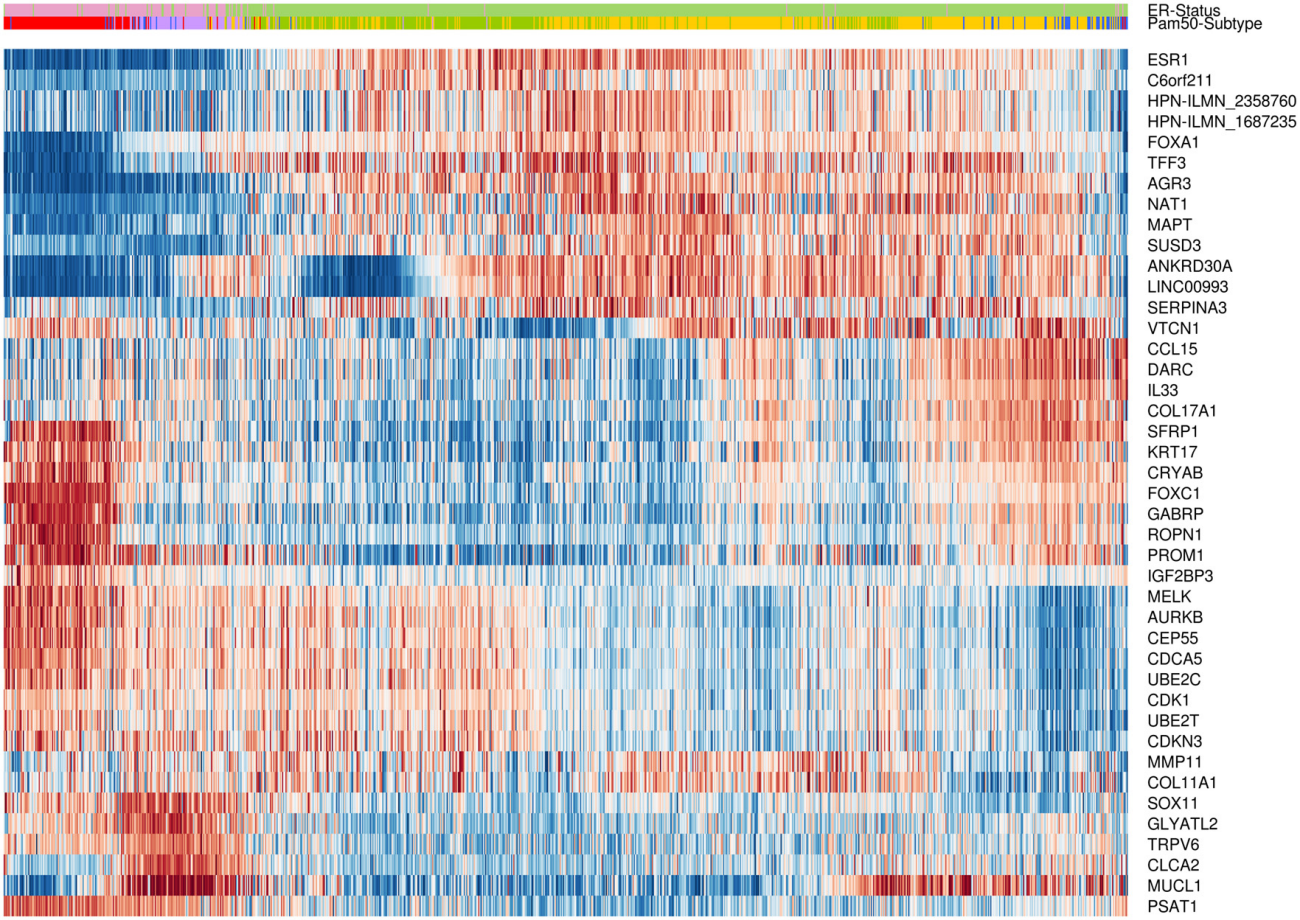
Gene expression patterns of the 42 probes selected using the CM1 score. The heat map diagram exhibit 42 probes (rows) and 997 samples (columns) from the discovery set ordered according to gene expression similarity, based on a memetic algorithm [27]. The labels highlighted on top show the sample distribution according to the ER positive and negative status. It also illustrates the original PAM50 subtypes luminal A (yellow), luminal B (green), HER2-enriched (purple), normal-like (blue), and basal-like (red) in the METABRIC discovery set. Two probes in the CM1 list refer to the same gene, *HPN*, which was then appended with the corresponding Illumina probe ID.

The effectiveness of the CM1 list for segregating the five subtypes is depicted in Fig 2. The figure shows the expression values of the top five negative and top five positive ranked probes for each subtype across 997 samples in the METABRIC discovery set. For instance, the ten probes selected for the basal-like subtype—the most representative class—expose a consistent separation between samples from this class and the remaining ones. The second heat map in Fig 3 illustrates the expression levels of unique probes from the CM1 list in the Illumina platform, in which rows represent probes and columns represent samples. Rows and columns were ordered according to gene expression similarity using a memetic algorithm [27]. This image also exposes the overall discriminative power of our list for distinguishing samples of the five subtypes.

**Fig 4:**
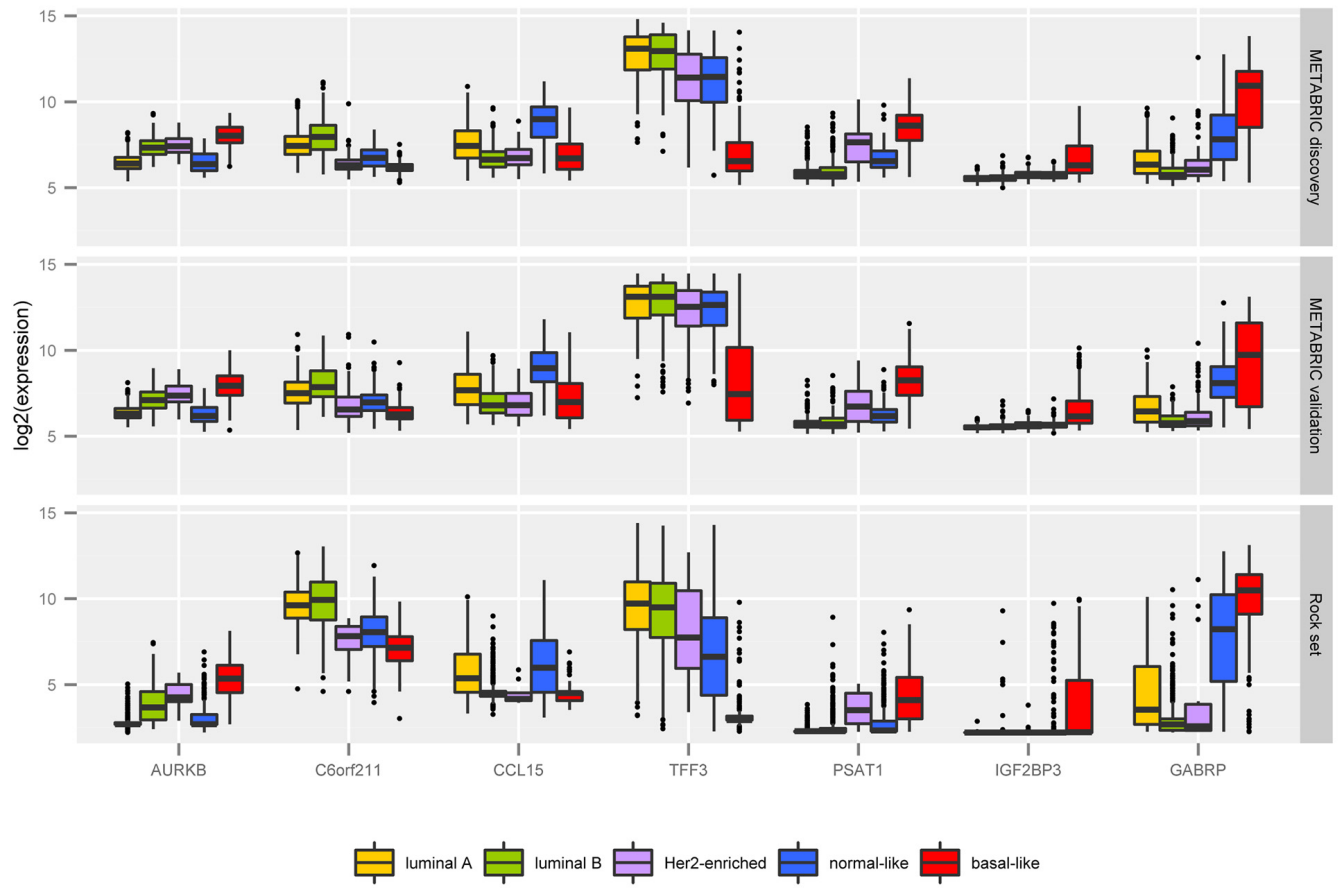
The mRNA log_2_ normalised expression values of 7 novel highly discriminative biomarkers across the five intrinsic subtypes in the METABRIC discovery and validation sets, and ROCK set. The box plot uncover the values of 997 samples in the METABRIC discovery set, 989 in the validation set, and 1570 in the ROCK test set.

A detailed description of our 42 probes in the context of the literature can be found in Supporting Information S1 Text. Among them we highlight seven, targeting the following transcripts: *AURKB*, *CCL15*, C6orf211, *GABRP*, *IGF2BP3*, *PSAT1*, and *TFF3*. Fig 4 shows the box plot of their expression levels across intrinsic subtypes in the METABRIC discovery and validation sets, and the ROCK set. We emphasized these transcripts due to the remarkable differential expression behaviour across the five classes. Besides, they are novel potential markers for breast cancer subtyping, not considered by Parker et al [16]. Box plots of expression levels for all transcripts in the CM1 list in the METABRIC discovery and validation and ROCK data sets are provided in Supporting Information S1 Fig. Even though those probes were selected from the METABRIC discovery set only, their variation across subtypes in the validation set and ROCK test set are also impressive.

### The ensemble of classifiers reveal high levels of agreement between CM1 and PAM50 lists

After applying the ensemble learning, several statistical measures were computed as referred in Materials and Methods. The main purpose of the statistics is to determine the performance of the 24 classification methods from the Weka software suite. In other words, we investigate the consistency of intrinsic subtype labels attributed by the majority of classifiers having as input either the CM1 or PAM50 lists. The quality of both lists was estimated according to the Cramer’s V statistic and the Average Sensitivity. Additionally, we computed the popular interrater reliability metric Fleiss’ kappa to establish the consensus of sample labelling across different classifiers. This metric was used to gauge the agreement among classifiers trained with CM1 and PAM50 lists against the original labels in the data sets, and between the labels assigned by the majority of classifiers using both lists. Ultimately, we applied the Adjusted Rand Index to quantify the agreement between pairs of samples that are either in the same class or in different classes according to both lists.

#### Average Cramer’s V statistic and Average Sensitivity to measure the performance of individual classifiers

We determined the performance of the ensemble learning (Supporting Information S2 Table, and S3 Table) with two measures: Cramer’s V statistic and Average Sensitivity (Table 3). Cramer’s V is used to measure the strength of association among variables in the row and column, given a contingency table (Tables 4, 5 and 6). The rows represent the original PAM50 labels and the columns the subtypes assigned by the majority of the classifiers in the ensemble. For instance, Cramer’s V statistic showed an average association between original and predicted subtypes of 0.73±0.06 and 0.63±0.04 in the METABRIC discovery and validation sets respectively with the CM1 list; and 0.75±0.06 and 0.64±0.04 with PAM50 list. Expanding the validation process using the ROCK test set, Cramer’s V ranged from 0.57±0.06 with the CM1, and 0.58±0.05 using PAM50 list.

**Table 3:** The ensemble learning overall performance on assigning labels to samples in the METABRIC discovery and validation sets, and ROCK test set.

	CM1 list		PAM50 list	
Dataset	CV	AS	CV	AS
**METABRIC discovery**	0.731 ± 0.057	0.763 ± 0.060	0.752 ± 0.064	0.781 ± 0.070
**METABRIC validation**	0.632 ± 0.036	0.641 ± 0.039	0.643 ± 0.041	0.650 ± 0.047
**ROCK test set**	0.571 ± 0.060	0.673 ± 0.077	0.578 ± 0.054	0.687 ± 0.081

Values are given as *average* ± *std. deviation*. CV- Cramer’s V; AS- Average Sensitivity

**Table 4:** Contingency tables for predicted labels using the 24 classifiers trained with the CM1 list.

	METABRIC discovery						METABRIC validation						ROCK test set					
	LA	LB	H	N	B	I	LA	LB	H	N	B	I	LA	LB	H	N	B	I
**LA**	435	19	2	2	0	8	252	2	0	0	0	1	452	122	2	0	0	17
**LB**	24	234	0	0	0	10	62	156	0	0	0	6	18	371	42	0	2	14
**H**	4	4	67	0	2	10	23	45	71	2	2	10	0	1	13	0	0	0
**N**	13	0	8	31	0	6	80	0	0	59	0	5	115	8	36	74	56	50
**B**	0	0	10	2	103	3	6	7	22	19	142	17	0	0	0	7	166	4

Rows contain labels assigned by the majority of classifiers trained with the CM1 list, while columns contain the the original METABRIC labels assigned using the PAM50 method. In this table, *LA* corresponds to luminal A, *LB* corresponds to luminal B, *H* to HER2-enriched, *N* to normal-like, and *B* to basal-like. Labels marked as *I* refer to inconsistent assignments; situations where the classifiers did not achieve the majority on attributing a subtype label.

**Table 5:** Contingency tables for predicted labels using the 24 classifiers trained with the PAM50 list.

	METABRIC discovery						METABRIC validation						ROCK test set					
	LA	LB	H	N	B	I	LA	LB	H	N	B	I	LA	LB	H	N	B	I
**LA**	440	17	1	1	0	7	254	0	0	0	0	1	530	46	2	0	0	15
**LB**	25	239	0	0	0	4	56	162	0	0	0	6	53	327	34	0	3	30
**H**	0	5	72	0	1	9	21	39	80	0	0	13	0	0	12	0	0	2
**N**	9	0	2	34	1	12	82	0	0	55	0	7	105	4	18	92	67	53
**B**	0	0	7	1	103	7	4	7	20	14	145	23	0	0	3	0	172	2

Rows contain labels assigned by the majority of classifiers trained with the PAM50 list, while columns contain the the original METABRIC labels assigned using the PAM50 method. In this table, *LA* corresponds to luminal A, *LB* corresponds to luminal B, *H* to HER2-enriched, *N* to normal-like, and *B* to basal-like. Labels marked as *I* refer to inconsistent assignments; situations where the classifiers did not achieve the majority on attributing a subtype label.

**Table 6:** Contingency tables for predicted labels using the 24 classifiers trained with CM1 and PAM50 lists.

	METABRIC discovery						METABRIC validation						ROCK Set					
	LA	LB	H	N	B	I	LA	LB	H	N	B	I	LA	LB	H	N	B	I
**LA**	450	15	0	4	0	7	390	14	1	4	0	14	550	8	0	10	0	17
**LB**	20	235	0	0	0	2	12	185	8	0	0	5	112	361	0	0	0	29
**H**	0	0	75	2	1	9	0	1	83	0	1	8	0	4	67	0	8	21
**N**	0	0	0	28	0	7	6	0	0	61	1	12	0	0	0	67	0	7
**B**	0	0	2	0	101	2	0	0	1	0	140	3	0	0	0	2	219	3
**I**	4	11	5	2	3	12	9	8	7	4	3	8	26	4	2	13	15	25

Rows contain the labels assigned by the majority of classifiers trained with the CM1 list, while columns contain labels assigned by the majority of classifiers trained with PAM50 list. In this table, *LA* corresponds to luminal A, *LB* corresponds to luminal B, *H* to HER2-enriched, *N* to normal-like, and *B* to basal-like. Labels marked as *I* refer to inconsistent assignments; situations where the classifiers did not achieve the majority on attributing a subtype label.

The Average Sensitivity statistic was used to characterize the average proportion of accurately labelled samples in each subtype. Considering the analysis with CM1 list, the measure was 0.76±0.06 in the METABRIC discovery set and 0.64±0.04 in the validation set; and with PAM50 list was 0.78±0.07 and 0.65±0.05, respectively. Likewise, the average sensitivity calculated for the ROCK test set was 0.67±0.07 using the CM1 and 0.69±0.08 with PAM50 list. A complete table containing the performance of all individual classification methods is available in the (Supporting Information S2 Table and S3 Table).

#### The levels of agreement explained by interrater reliability metric Fleiss’ kappa

Fleiss’ kappa was computed to assess the reliability of agreement between two raters, as displayed in Table 7. We initially compared the agreement *Among classifiers* which indicates the overall performance of classifiers alone. We then compared *Predicted vs Original*, that is, the agreement between subtypes assigned by the majority of classifiers using CM1 and PAM50 lists compared to the original PAM50 labels in the METABRIC discovery and validation sets, and ROCK test set. We also calculated the kappa between labels attributed by the majority of classifiers using both lists, *CM1 vs PAM50*. We refer to the Materials and Methods section for an interpretation of *κ* values. For instance, the high levels of agreement between two raters reflect more than what would be expected by chance.

**Table 7:** Agreement of the 24 classifiers on assigning labels to samples in the data sets measured by Fleiss’ kappa statistic.

		METABRIC		ROCK
		discovery	validation	test set
**Among classifiers**	CM1	0.73	0.753	0.626
	PAM50	0.724	0.729	0.59
**Predicted vs. Original**	CM1	0.814	0.596	0.591
	PAM50	0.84	0.618	0.641
**CM1 vs PAM50**		0.859	0.832	0.804

Rows entitled *Among classifiers* indicate agreement of classifiers alone, not considering the labels. *Predicted vs Original* show the agreement between the mostly predicted and initial labels of samples (PAM50 method). Finally, rows entitled *CM1 vs PAM50* contain the agreement between the mostly predicted labels using the CM1 and PAM50 lists with the ensemble learning.

Considering the agreement of the ensemble of classifiers, there was a *substantial agreement* in both METABRIC discovery and validation sets, and ROCK test set (Table 7). Fleiss’ kappa was 0.73, 0.75 and 0.63 with the CM1 list for METABRIC discovery, validation and ROCK data sets, respectively. Values obtained with the PAM50 list were 0.72, 0.73 and 0.6, respectively. By comparing the subtypes predicted by the majority of classifiers and original PAM50 labels, there was an *almost perfect agreement* with CM1 (*κ* = 0.81) and PAM50 (*κ* = 0.84) lists in the discovery set. In the validation and ROCK sets, on the other hand, labels showed only a *moderate agreement* for both lists (*κ* ≃ 0.6). Strikingly, the Fleiss’ kappa between subtypes predicted using the CM1 and PAM50 lists (*κ* = 0.86, 0.83, and 0.8 in the METABRIC discovery, validation, and ROCK sets, respectively) revealed an *almost perfect agreement*. This statistical measure confirm our visual analysis of the contingency tables as they find strong relationship across the subtype labels in each data set. A detail of the agreement among classifiers by intrinsic subtype is shown in (Supporting Information S4 Table).

#### The agreement according to the Adjusted Rand Index

The agreement between the different sample labellings was also scrutinized using the Adjusted Rand Index measure (Table 8). The values obtained with the CM1 list were 0.757 in the METABRIC discovery and 0.426 in the validation sets, and 0.453 in the ROCK test set. For PAM50 list, the values were 0.792, 0.457 and 0.507, respectively. Similar to Fleiss’ kappa, the agreement between labels predicted with CM1 and PAM50 lists is higher than the agreement with the original labels. The Adjusted Rand Index values were 0.822, 0.788 and 0.642 for the three data sets, respectively. The numbers obtained with this measure also revealed remarkable concordance of CM1 and PAM50 lists assigned labels.

**Table 8:** Agreement measured by the Adjusted Rand Index between different samples’ labellings.

	METABRIC		ROCK
	discovery	validation	test set
**CM1**	0.757	0.426	0.453
**PAM50**	0.792	0.457	0.507
**CM1-PAM50**	0.822	0.788	0.642

This contains the agreement between the original and predicted labels of samples in the discovery and validation sets. *CM1-METABRIC* refers to agreement between the labels predicted by the majority of classifiers trained with the CM1 list and the original METABRIC labels; *PAM50-METABRIC* is the agreement between labels predicted by the majority of classifiers trained with the PAM50 list and original METABRIC labels; and *CM1-PAM50* is the agreement between predicted labels using both lists.

### The use of an ensemble learning with the CM1 list improves the subtype distribution in the METABRIC and ROCK data sets

**Fig 5:**
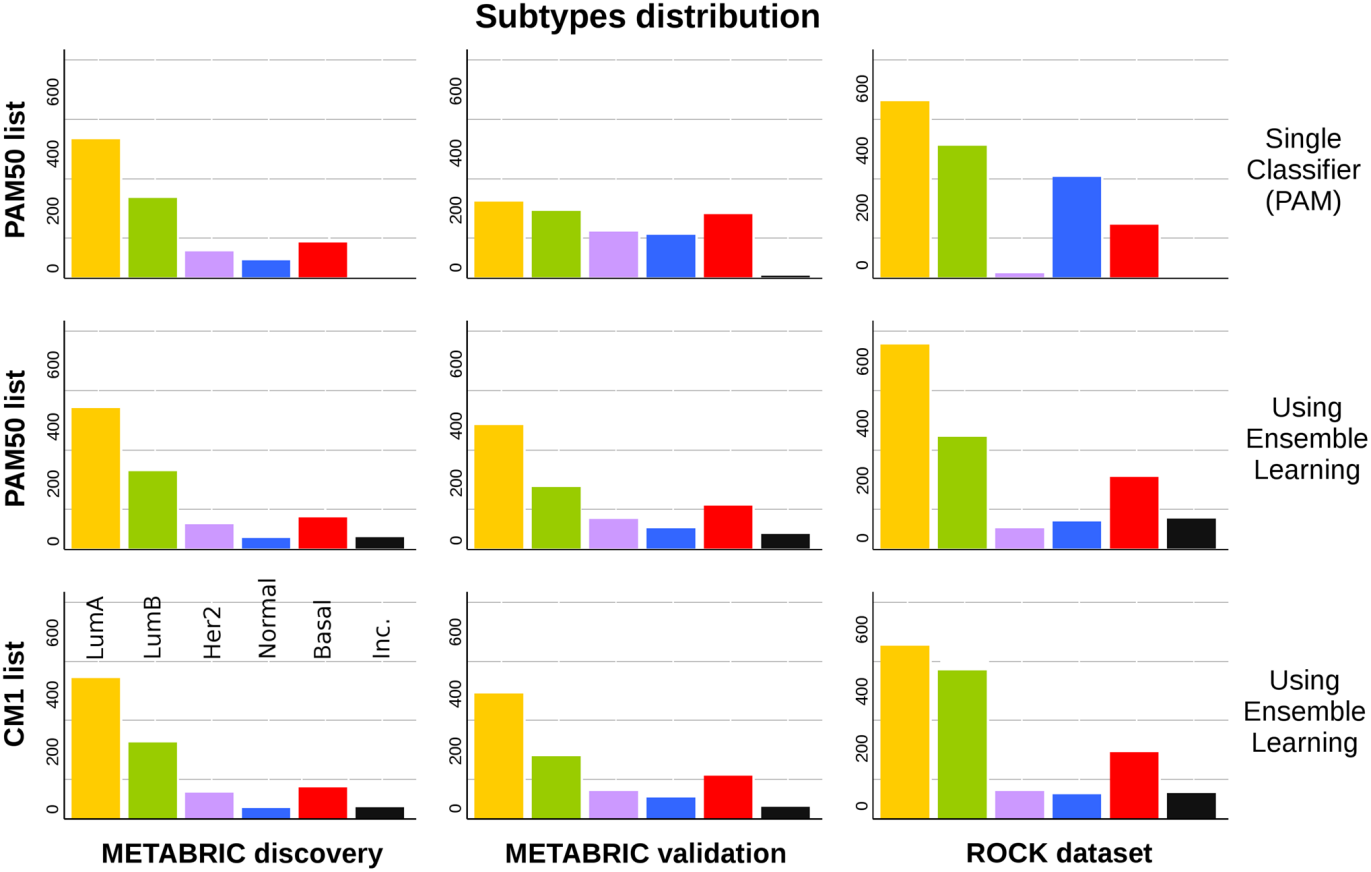
Class distribution in the METABRIC discovery and validation sets, and in the ROCK set. The bars represent the number of samples in each breast cancer subtype. In the first row, the labels refer to the original assignment using the PAM50 method. The following rows show the new labels attributed using an ensemble of 24 classifiers with PAM50 and CM1 lists, respectively. Samples were classified as *inconsistent* if there was no consensus between the majority of classifiers as to what should be the correct subtype.

The number of samples in each original PAM50 subtype is markedly different across the METABRIC sets (Fig 5). In the discovery set, there is a clear abundance of luminal A and B subtypes, precisely 73.62% of all samples. In contrast, the proportion of luminals in the validation set is only 48.14%. The ratio of luminal A to luminal B samples changed from 1.74 in the discovery to 1.14 in the validation set. However, when the CM1 or PAM50 lists are used in conjunction with the ensemble of classifiers, samples in the discovery and validation sets are more homogeneously distributed. The percentage of samples in the discovery set labelled as luminal A and B using CM1 and PAM50 lists are 73.53% and 73.72%, respectively. These proportions match the original number (73.62%). On the other hand, in the validation set the CM1 and PAM50 lists assigned a total of 64% and 63.19% luminal samples, against the 48.14% previously mentioned. The distribution of subtypes also become more similar to the discovery set. Likewise, ROCK test set also changed the pattern of class distribution after the performance of the ensemble of classifiers. The differences in class distributions might not be attributed to the randomisation procedure used by the studies as the performance of the ensemble of classifiers with both lists reconcile the distribution of subtypes.

**Fig 6:**
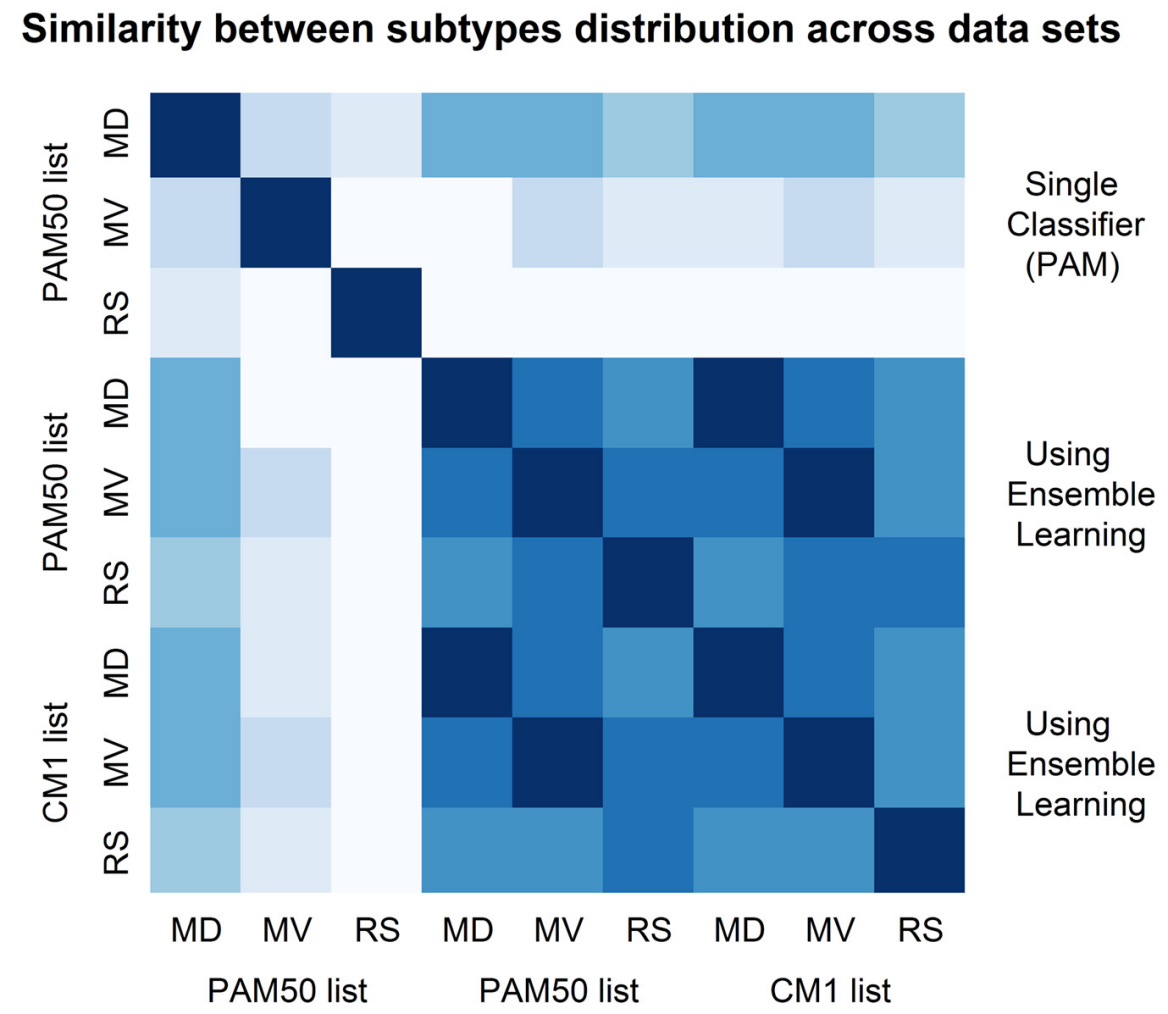
Similarity between subtypes distribution in the METABRIC discovery and validation sets, and in the ROCK set. The image shows the similarity between the subtypes distribution for METABRIC discovery (MD) and validation (MD) sets, and ROCK test set (RS). The labels were assigned in the original data sets using the PAM50 method, and relabelled in this study with an ensemble learning using PAM50 and CM1 lists. The similarity is measured using the square root of the Jensen-Shannon divergence. Darker shades represent more similar distributions, while lighter shades refer to divergent patterns. The diagonal shows the darkest color as each data set is the closest to itself. According to this image, labels assigned using an ensemble learning with CM1 and PAM50 lists are highly similar, and both exhibit lower levels of agreement with the original labels assigned using a single classifier (PAM), or PAM50 method.

We summarize the similarities and differences in subtypes distribution (graphically displayed in Fig 5) by computing the square root of the Jensen-Shannon divergence [225]. This is a true metric of distance between probability distributions. Its plot in Fig 6 shows the similarity between all possible pairs of data sets based on their distribution of subtype labels (Supporting Information S4 Table). It can be observed that the original labels are the most divergent ones, especially in the METABRIC validation and ROCK test sets. The high similarity of samples distribution among subtypes based on the assignments with CM1 or PAM50 lists is evident. Such similarity was not expected for the ROCK set as the ensemble of classifiers was trained with METABRIC discovery (Illumina platform data) and tested in the ROCK set (Affymetrix platform data). The limited number of probes matching Illumina and Affymetrix in both lists (as described in Materials and Methods) seems not to affect the performance of the ensemble learning. Yet the divergences in the original class distributions might not be attributed to the randomisation procedure used by the consortium. These results point out to the relative strength and robustness of a set of classifiers compared to single methods to predict breast cancer subtype labels. They also indicate that there is an issue to be considered by researchers when using the original PAM50 labels from the METABRIC study for analysing data and building predictive models.

### Breast cancer intrinsic subtypes show different clinical markers distribution and survival curves

**Fig 7:**
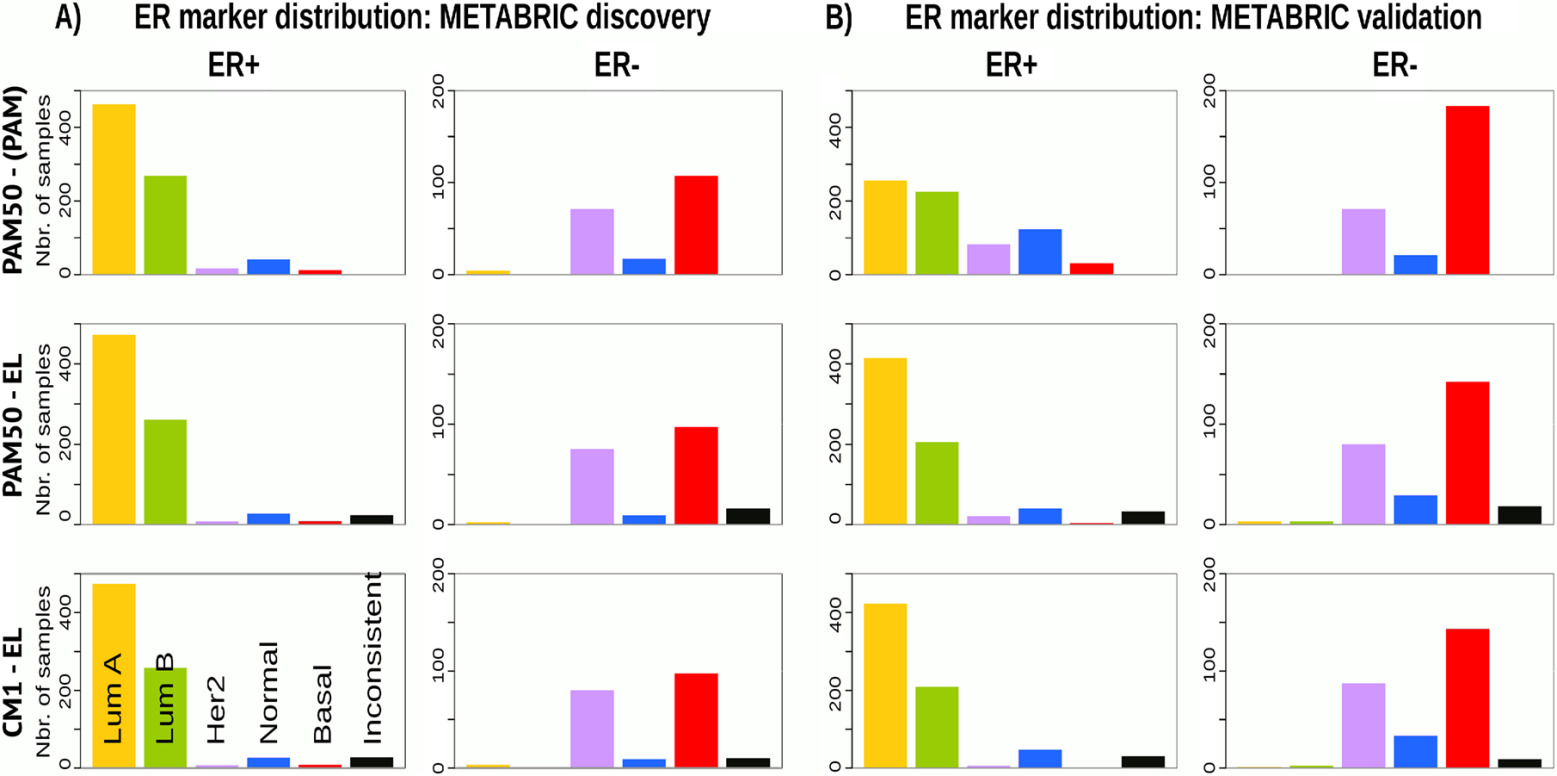
ER marker distribution across subtypes in the METABRIC data sets. (A) Discovery and (B) Validation. The bars represent the number of samples with ER positive and negative in the five intrinsic subtypes, based on the patients’ clinical information. The top row is based on the original subtype labels obtained with the PAM50 list and a single classifier (PAM). Middle and bottom rows are based on the labels obtained by Ensemble Learning using the PAM50 and CM1 lists, respectively.

**Fig 8:**
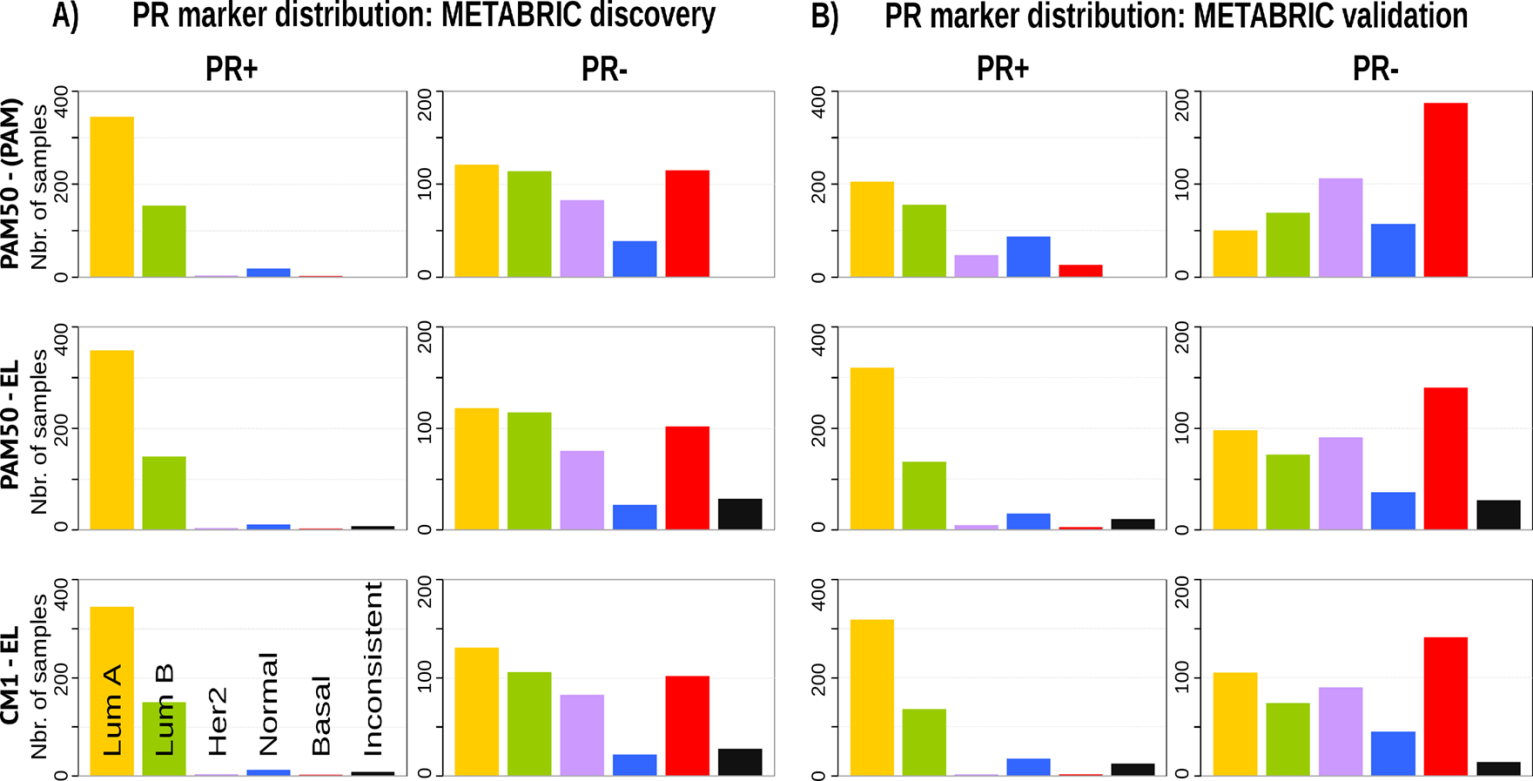
PR marker distribution across subtypes in the METABRIC data set. (A) Discovery and (B) Validation. The bars represent the number of samples with PR positive and negative distributed in the five intrinsic subtypes, based on the patients’ clinical information. The top row is based on the original subtype labels obtained with the PAM50 list and a single classifier (PAM). Middle and bottom rows are based on the labels obtained by Ensemble Learning using the PAM50 and CM1 lists, respectively.

**Fig 9:**
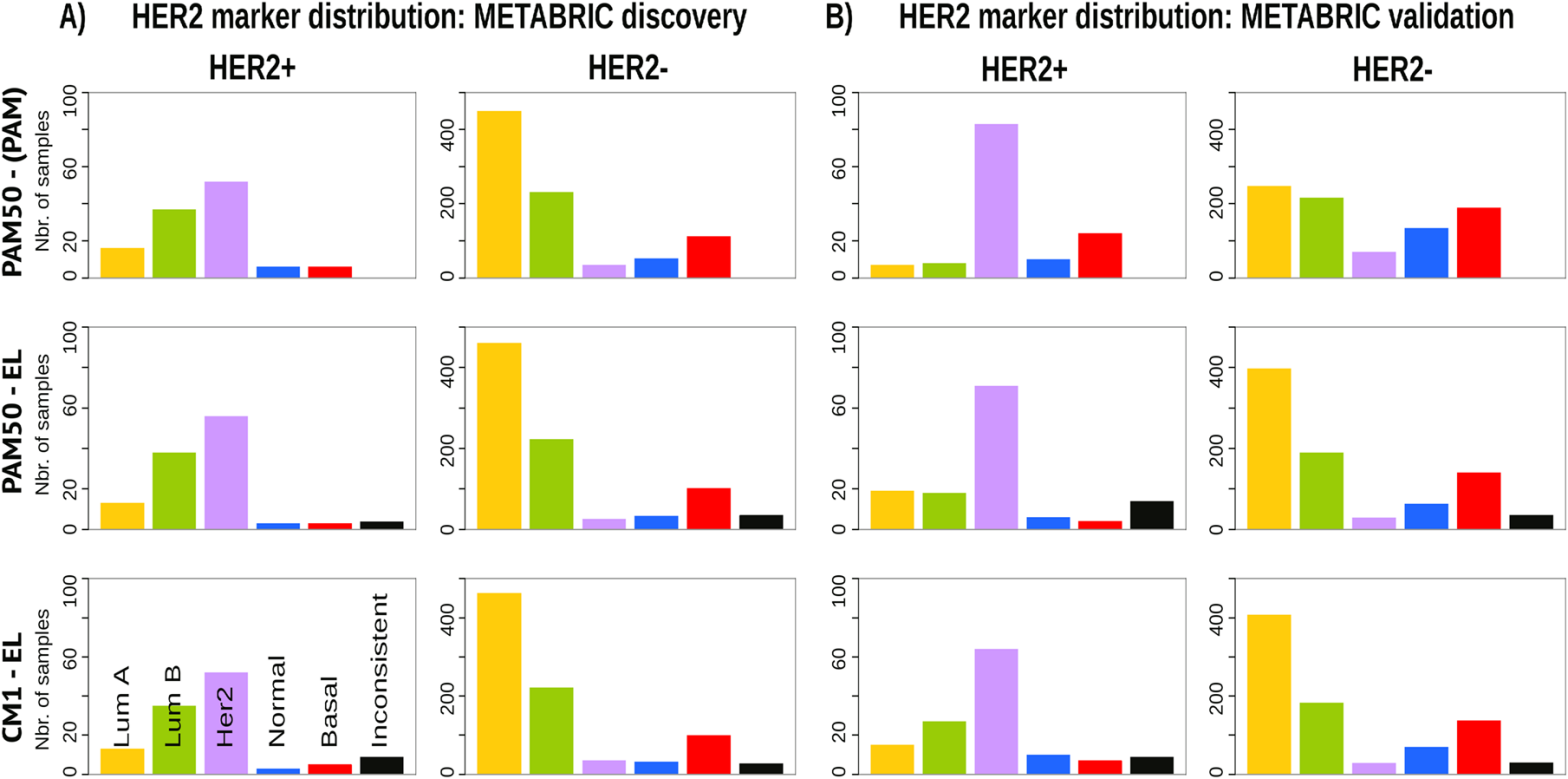
*HER2* distribution across subtypes in the METABRIC data sets. (A) Discovery and (B) Validation. The bars represent the number of samples with *HER2* amplification (positive or negative) for each intrinsic subtype based on the patients’ clinical information. The top row is based on the original subtype labels obtained with the PAM50 list and a single classifier (PAM). Middle and bottom rows are based on the labels obtained by Ensemble Learning using the PAM50 and CM1 lists, respectively.

Given the heterogeneity among breast cancer patients and the intricate assignment of PAM50 labels in the original METABRIC data set, we further investigated whether significant differences exist in the analysis of current clinical markers (ER, PR and HER2). Figs 7, 8 and 9 show, respectively, the distribution of the ER, PR and *HER2* across intrinsic subtypes in the METABRIC discovery and validation sets, considering the original PAM50 labels and the labels assigned by ensemble of classifiers using CM1 and PAM50 lists. The new subtype labelling markedly improves the status of the clinical markers in the METABRIC data set. For instance, the ER marker distribution across subtypes shows an important decrease in the number of HER2-enriched and basal-like samples that are ER-positive according to the original PAM50 labels. The PR marker, likewise, varies the distribution when predicted labels based on the ensemble of classifiers using either CM1 and PAM50 list are compared with the original labels. *HER2* amplification has a particular behaviour across all subtypes. Under the new subtype labels, the distribution of the three clinical markers becomes more consistent with what is expected according to the literature for each class: luminal A (ER+ and/or PR+, *HER2*−); luminal B (ER+ and/or PR+, *HER2*±); HER2-enriched (ER−, PR− and *HER2*+); and basal-like (ER−, PR−, *HER2*−) [226].

**Fig 10:**
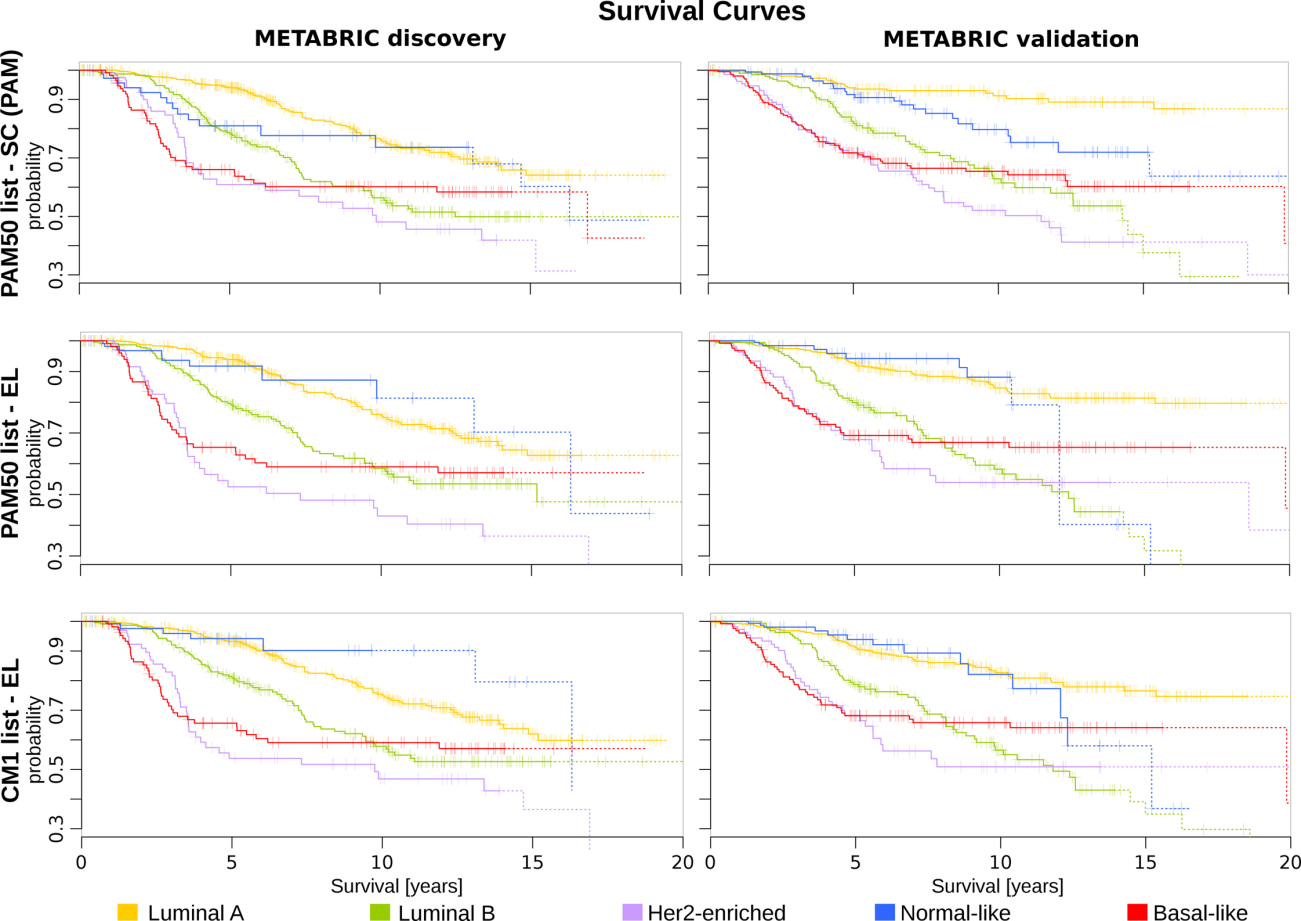
The survival curves for METABRIC discovery and validation sets. The survival curves for each breast cancer subtype are generated using Cox proportional hazards model based on the grade and size of the tumour, patient’s age, number of lymph nodes positive and ER status. Each curve represents the survival probability at a certain time after the diagnosis. Ticks on the curve correspond to the observations of patients who are still alive, while drops indicate the death. The probability curves based on the last 10 observations are plotted in dash. The top row is based on the original subtype labels obtained with the PAM50 list and a single classifier (PAM). Middle and bottom rows are based on the labels obtained by Ensemble Learning using the PAM50 and CM1 lists, respectively.

Subsequently, we illustrate the survival curves for all breast cancer subtypes using Cox proportional hazards model, as described in Materials and Methods. The curves were plotted based on the original PAM50 labels and those assigned by the majority of classifiers. For generating the survival curves, we included the most relevant clinical variables as covariates: grade, size, age at diagnosis, number of lymph nodes positive, and ER status (immunohistochemistry) [27]. This analysis revealed different curves in the METABRIC discovery and validation sets (Fig 10). For instance, luminal B and basal-like subtypes show a similar pattern across data sets. Luminal A, HER2-enriched and normal-like, on the other hand, have a more consistent survival pattern when the CM1 and PAM50 lists are used in conjunction with the ensemble learning. Taken as a whole, the results of this section support the increased robustness of labels assigned by the ensemble of classifiers with the CM1 or PAM50 lists, and point out to inconsistencies in the original subtype assignment in the METABRIC study.

## Discussion

In this study, we exposed the power of the CM1 list for improving the breast cancer subtype prediction in the METABRIC and ROCK data sets. The CM1 score portrayed 30 novel genes as potential biomarkers, along with 12 well-established markers shared between CM1 and PAM50 lists. The 42 biomarkers have a great potential to differentiate breast cancer intrinsic subtypes. Among them, *AGR3, HPN, ANKRD30A, AURKB, PROM1, VTCN1, CRYAB, CDK1, CDKN3, SERPINA3, SOX11, TRPV6, CLCA2, MUCL1, COL11A1, DARC, TFF3, IGF2BP3, IL33, SUSD3, PSAT1*, and *GABRP* are reported in different studies associated with breast cancer; however not in the context of subtype differentiation. Noteworthy, the CM1 list revealed a set of probes for which little literature exists in relation to breast cancer subtypes: *CDCA5, CCL15, COL17A1, GLYATL2, ROPN1*, LINC00993 and C6orf211. Their expression levels vary across different subtypes, and are yet a new avenue to be explored. We also emphasize the 12 common genes (*CEP55, ESR1, FOXA1, FOXC1, KRT17, MAPT, MELK, MMP11, NAT1, SFRP1, UBE2C*, and *UBE2T*) due to their important role for breast cancer disease and intrinsic subtyping.

Within the application of an ensemble of classifiers, CM1 and PAM50 lists showed concordant predictive power for disease subtyping. In fact, there was an *almost perfect agreement* between the labellings obtained with the majority of classifiers using both lists; however different from the original labels. In this study, we want to highlight the weakness of relying in a single method to assign subtypes labels, as opposed to the power and robustness of an ensemble learning. We therefore discourage label assignments based on a single classifier and also suggests a thorough review of those intrinsic subtypes given the importance of such data sets to breast cancer research. The results indicate that there is an issue to be considered by researchers when using the original PAM50 labels for analysing data. The use of incorrect labels would lead to a plethora of misguided and misleading results by other investigators that use METABRIC or ROCK data sets.

In spite of luminals sharing the same origin and large molecular commonalities [227, 228], the ensemble of classifiers accurately predicted luminal samples in the METABRIC data set, and showed some ambiguity on assigning the subtype A or B for a small number of samples, specially in the ROCK data set. This may be a consequence of the reduced number of probes matching across Illumina and Affymetrix platforms. HER2-enriched notably improved label consistency in the ROCK data. Furthermore, the normal-like tumours received more often contradictory and inaccurate subtype labelling among both data sets. The poor overall outcome for this subtype is supported by the discussion that normal-like is an artefact of sample processing with high contamination of normal breast tissue [13, 16, 229]; however, still crucial to be elucidated. Ultimately, the basal-like subtype maintained the classification with a unique profile, markedly divergent from other subtypes [21, 22, 230]; even though this group has recently been partitioned into new fundamental classes [9, 10].

Overall, the new intrinsic subtype labels based on the CM1 list and ensemble learning revealed more accurate distributions of clinical markers (ER, PR and HER2) and survival curves, when compared to the original PAM50 labels in the METABRIC cohort and ROCK test set. Interestingly, the CM1 list shows *ESR1* (ER) among the 42 probes, but brings other independent genes that are also relevant for overall predictions. Robust data sets like METABRIC have contributed to the understanding of breast cancer disease in terms of its molecular complexity and intrinsic alterations. The main limitations of the research in the field, nevertheless, is the uncertainty in the exact classification of intrinsic subtypes; over and above the discovery of molecular signatures and standard clinical biomarkers. Under consideration, a consistent taxonomy needs yet to be established prior to implementation in clinical practice. Additional research involving the genome, transcriptome, proteome, and epigenome, will lastly portray a true landscape of subtypes and contribute to breast cancer management.

## Supporting Information

S1 Text CM1 list and literature review. The document shows the CM1 probe list along with an extensive literature review. The 42 CM1 biomarkers revealed a great potential to differentiate breast cancer intrinsic subtypes in the METABRIC and ROCK data sets. The 30 novel markers and 12 well-established genes vary the expression levels across different subtypes. The vast majority has been associated with breast cancer disease, either included or not in the subtyping context.
(PDF)

S1 Fig The mRNA log2 normalised expression values of 42 probes in the CM1 list across the five intrinsic subtypes in the METABRIC discovery and validation sets, and ROCK set. Box plots illustrating the expression levels for all selected transcripts in the CM1 list in the METABRIC discovery and validation sets, and ROCK test set. The figure shows the probes differential behaviour across breast cancer intrinsic subtypes.
(TIFF)

S1 Table The CM1 score calculated for each breast cancer subtype. Table listing the CM1 score used to rank the set of 48803 probes for each of the five breast cancer subtypes in the METABRIC discovery data set. In each case, we selected the top 10 highly discriminative probes (5 with the greatest positive CM1 score values—indicating up-regulated probes relative to the other subtypes, and 5 with the smallest negative values—representing down-regulation).
(XLSX)

S2 Table The performance of the classifiers using the CM1 list. Table describing the performance of each classifier on the METABRIC discovery and validation sets, and ROCK test set using the CM1 list. It shows the percentage of correctly, incorrectly and not classified samples, Fleiss Kappa index, Cramer’s V, Average Sensitivity, and other values for classification. The 24 classifiers from the Weka software suite are also listed. The labels predicted by each classifier for all samples using CM1 list are defined as: 1—luminal A, 2—luminal B, 3—HER2-enriched, 4—normal-like, 5—basal-like. Count of predicted labels was obtained with the consensus of the majority of classifiers.
(XLSX)

S3 Table The performance of the classifiers using the PAM50 list. Table describing the performance of each classifier on the METABRIC discovery and validation sets, and ROCK test set using the PAM50 list. It shows the percentage of correctly, incorrectly and not classified samples, Fleiss Kappa index, Cramer’s V, Average Sensitivity, and other values for classification. The 24 classifiers from the Weka software suite are also listed. The labels predicted by each classifier for all samples using CM1 list are defined as: 1—luminal A, 2—luminal B, 3—HER2-enriched, 4—normal-like, 5—basal-like. Count of predicted labels was obtained with the consensus of the majority of classifiers.
(XLSX)

S4 Table The agreement between sample labelling with Fleiss’ Kappa measure and the Jensen-Shannon divergence of two probability distributions. Table containing the Fleiss’ Kappa agreement of labels for the METABRIC discovery and validation sets, and ROCK test set. It shows the overall agreement *Among classifiers* using CM1 and PAM50 lists, as well as the agreement for each subtype. The *predicted—original* are described in the table and contain the agreement between the mostly predicted and initial labels of samples; whereas the *CM1—PAM50* show agreement between labels assigned by the majority of classifiers using CM1 and PAM50 lists. The file also has the Jensen-Shannon divergence between two probability distributions. Numbers represent the similarity between subtypes distribution for METABRIC discovery and validation sets, and ROCK test set. The similarity is measured using the square root of the Jensen-Shannon divergence.
(XLSX)
