# Supplementary material for: The Discovery of Novel Biomarkers Improves Breast Cancer Intrinsic Subtype Prediction and Reconciles the Labels in the METABRIC Data Set
Source: PLoS One. 2015 Jul 1;10(7):e0129711. doi: 10.1371/journal.pone.0129711 (PMC4488510; doi:10.1371/journal.pone.0129711)
Supplement: S1 Text — The document shows the CM1 probe list along with an extensive literature review. The 42 CM1 biomarkers revealed a great potential to differentiate breast cancer intrinsic subtypes in the METABRIC and ROCK data sets. The 30 novel markers and 12 well-established genes vary the expression levels across different subtypes. The vast majority has been associated with breast cancer disease, either included or not in the subtyping context. (PDF) [file pone.0129711.s001.pdf]

Illumina Probe_ID	Symbol	Luminal A	Luminal B	Her2 enriched	Normal like	Basal like	Gene and Protein Review
ILMN_1684217	AURKB	-					Aurora kinase B (*AURKB*; also known as *Aik2, AIK2, AIM1, AIM-1, ARK2, ARK-2, AurB, aurkb-sv1, aurkb-sv2, Aurora/IPL1-related kinase 2, Aurora-and IPL1-like midbody-associated protein 1, Aurora kinase B, Aurora-related kinase 2, IPL1, Serine/threonine-protein kinase 12, Serine/threonine-protein kinase aurora-B, STK-1, STK12, STK5*) encodes a protein member of the aurora kinase subfamily of serine/threonine kinases that function as a regulator of the centrosome cycle and mitotic spindle assembly. The protein AURKB and interacting proteins play an important role in chromosome condensation, segregation and cytokinesis and, consequently, in ploidy maintenance during cell division. Mitotic deregulations may contribute significantly to cell division errors and development of aggressive tumour cells [1,2]. AURKB signalling was also linked to breast cancer and associated to poor prognosis [3]. This gene has been a target of inactivation via different studies and mechanisms [4-13].
ILMN_1683450	CDCA5	-					Cell division cycle associated 5 (*CDCA5*; also known as *Cell division cycle-associated protein 5, MGC16386, p35, Sororin, SORORIN*) is important for sister chromatid cohesion during mitosis, stabilizing proper chromatin association during G2 phase. The protein is also needed for efficient repair of DNA double-strand breaks and for stable presence of normal amounts of chromatin-bound cohesin population [14,15]. Carretero et al [16] reported that “the reduced accumulation of AURKB at the inner centromere in cells that lack PDS5B impairs its error correction function, promoting chromosome mis-segregation and aneuploidy”. Although systematic studies showed the up-regulation of this gene in a great majority of lung cancers, its involvement in breast cancer disease requires further investigation. The protein CDCA5 confers a potential diagnostic molecule and therapeutic target for promising strategies in new drug development [17].
ILMN_1747016	CEP55	-					Centrosomal protein 55kDa (*CEP55*; also known as *C10orf3, Centrosomal protein of 55 kDa, Cep55, CT111, FLJ10540, Up-regulated in colon cancer 6, URCC6*) encodes a mitotic phosphoprotein that acts in mitotic exit and cytokinesis. Both down- and up-regulation of *CEP55* causes a cytokinesis defect [18]. The gene expression variance, nonetheless, is not affected by hormone receptors such as oestrogen and progesterone or expression patterns of *ERBB2*. The centrosomal protein is detected in a wide variety of tumour cell lines and is considered as a novel breast tumour-associated antigen [19]. It was also reported that *CEP55* have genomic alternation in a comparison of pre-invasive ductal carcinoma in situ (DCIS) to invasive ductal carcinoma (IDC) by Colak et al. [20] and it was predictive of prognosis in ER-positive patients [21].
ILMN_2212909	MELK	-					The maternal embryonic leucine zipper kinase (*MELK*; also known as *hMELK, hPK38, HPK38, KIAA0175, Maternal embryonic leucine zipper kinase, Protein kinase PK38*) acts as a regulator of various processes such as cell cycle control, self-renewal of stem cells, apoptosis, and splicing regulation. The encoded protein physically interacts, phosphorylates and inhibits BCL2L14, repressing a pro-apoptotic member of the Bcl-2 family. The protein also mediates phosphorylation of CDC25B, regulating the entry into mitosis. In addition, MELK inhibits the spliceosome assembly during mitosis by phosphorylating ZNF622 and contributes to induce other apoptosis signalling regulation. The protein kinase is a promising molecular target for the treatment of breast cancer [22] as the up-regulation is linked to poor prognosis [23-27]. Finally, it has been suggested that paclitaxel may attenuate the expression of MELK [28].
ILMN_1714730	UBE2C	-					Ubiquitin-conjugating enzyme E2C (*UBE2C*; also known as *dJ447F3.2, UbcH10, UBCH10, Ubiquitin carrier protein C, Ubiquitin-conjugating enzyme E2 C, Ubiquitin-protein ligase C*) encodes a member of the E2 ubiquitin-conjugating enzyme family. The ubiquitin modification in proteins is an important cellular mechanism of homoeostasis and fate [29]. UBE2C is required for cell cycle progression and checkpoint control through targeted degradation of short-lived proteins, the mitotic cyclins. Aberrations in this pathway is implicated in cancer progression and, importantly, in the pathogenesis of breast cancer [30,31]. The *UBE2C* up-regulation is also normally linked to high tumour grade and poor prognosis [32,33].

Illumina Probe_ID	Symbol	Luminal A	Luminal B	Her2 enriched	Normal like	Basal like	Gene and Protein Review
ILMN_1796059	ANKRD30A	+				-	Ankyrin repeat domain 30A (*ANKRD30A*; also known as *Ankyrin repeat domain-containing protein 30A, NY-BR-1, Serologically defined breast cancer antigen NY-BR-1*) is an antigen expressed in mammary glands, primary and metastatic breast carcinomas [34-36]. Interestingly, *ANKRD30A* is almost expressed exclusively in breast epithelium; with exception of testis and sweat gland tumours. Despite the insufficient knowledge about the biology and function of the gene, the tissue specificity may be useful for the diagnosis of breast carcinomas [34,37-39]; and a potential target for treatment (immunotherapy) [40,41].
ILMN_1651329	LINC00993	+					The long intergenic non-protein coding RNA 993 (LINC00993) matches a region in the chromosome 10 very close to *ANKRD30A*; and contains a SNP (rs77587276) variant. The region requires further investigation as it covers relevant probes associated with breast cancer disease; markedly, up-regulated in luminal subtype.
ILMN_2310814	MAPT	+					The microtubule-associated protein tau (*MAPT*; also known as *DDPAC, FLJ31424, FTDP-17, MAPTL, MGC138549, Microtubule-associated protein tau, MSTD, MTBT1, MTBT2, Neurofibrillary tangle protein, Paired helical filament-tau, PHF-tau, PPND, tau, TAU*) undergoes alternative splicing, originating several mRNA transcripts. The isoforms differ by having variant conserved repeat motifs in the microtubule-binding domain, and insertions in the N-terminal projection domain. Although the function of each isoform is unknown, the protein binds to both the outer and inner surfaces of microtubules, organizing the tubulin assembly and microtubule stabilization [42]. In breast cancer, *MAPT* expression is high in ER-positive low grade compared to ER-negative high grade tumours [43]. Additionally, the gene up-regulation is correlated with favourable prognosis [44] and, at the same time, associated with resistance to taxanes, paclitaxel and docetaxel [42,45-49].
ILMN_1728787	AGR3	+	+	-		-	The anterior gradient 3 (*AGR3*; also known as *AG3, AG-3, Anterior gradient protein 3 homolog, BCMP11, Breast cancer membrane protein 11, HAG3, hAG-3, PDIA18, UNQ642/PRO1272*) functionality has been defined in breast cancer cells as involved in hormone responsiveness, cell adhesion, migration, and metastasis. This gene encodes a membrane protein with a potential role in tumorigenesis by interacting with metastasis-associated genes [50,51].
ILMN_1688071	NAT1	+		-			The enzyme encoded by N-acetyltransferase 1 (*NAT1*; also known as *AAC1, Arylamide acetylase 1, Arylamine N-acetyltransferase 1, MNAT, Monomorphic arylamine N-acetyltransferase, N-acetyltransferase type 1, NAT-1, NATI*) acts metabolizing drugs and other xenobiotics, and functions in folate catabolism [52,53]. Kim et al. (2008) reported the hypomethylation of the *NAT1* promoter region resulting in aberrant mRNA expression levels, with overexpression of the gene in breast carcinomas. Likewise, new insights into the associations of SNPs in the coding and control regions of *NAT1* have been described and suggested as a potential susceptibility biomarker for the disease [53,54].

Illumina Probe_ID	Symbol	Luminal A	Luminal B	Her2 enriched	Normal like	Basal like	Gene and Protein Review
ILMN_1729216	CRYAB		-				Crystallin, alpha B (*CRYAB*; also known as *Alpha(B)-crystallin, Alpha-crystallin B chain, CRYA2, CTPP2, Heat shock protein beta-5, HspB5, HSPB5, Renal carcinoma antigen NY-REN-27, Rosenthal fiber component*) is a member of the small heat shock protein (sHSP; also known as the HSP20) family, all of which share a common C terminal motif – the alpha crystallin domain. The protein CRYAB acts as molecular chaperones induced by ubiquitous stress and up-regulated by heat, radiation, oxidative stress and anticancer drugs. Other additional functions of alpha crystallins are the autokinase activity, participation in the intracellular architecture, and the control of large soluble protein aggregates. Additionally, CRYAB shows redundancy in interacting with various apoptosis pathways at multiple levels [55]; besides plays a critical role in vasculature homeostasis and angiogenesis [56]. The protein is expressed widely in many tissues and organs [57]. In breast cancer, *CRYAB* is usually high differentially expressed in invasive tumours when compared to normal breast tissue specimens [55] and might be involved in chemotherapy response [58].
ILMN_1666845	KRT17		-				The protein encoded by keratin 17 (*KRT17*; also known as *39.1, CK-17, Cytokeratin-17, K17, Keratin, type I cytoskeletal 17, Keratin-17, PC, PC2, PCHC1*) plays a role in the formation and maintenance of various epidermal appendages, as the nail bed, hair follicle, and sebaceous glands. KRT17 regulates other protein synthesis and epithelial cell growth. In addition, the protein is a marker of basal cell differentiation as an attribute of a certain type of "stem cells". In the context, immunohistochemical studies revealed that basal-like breast tumours present *KRT17* up-regulation, with levels associated with a poor clinical outcome [59]. This gene is also up-regulated in primary *BRCA1* mutant breast tumours; fact consistent with the reported connection between *BRCA1* mutation and basal-like subtypes [60].
ILMN_1786720	PROM1*		-				Prominin 1 (*PROM1*; also known as *AC133, Antigen AC133, CD133, CORD12, MCDR2, MSTP061, Prominin-1, Prominin-like protein 1, PROML1, RP41, STGD4*) encodes a transmembrane glycoprotein, often expressed on adult stem cells. The protein plays an essential role in maintaining stem cell properties by suppressing differentiation. Aberrant expression *PROM1* is also associated with several types of cancer. In breast cancer, *PROM1* overexpression is positively related to tumour size, stage, and lymph node metastasis in invasive tumours [61]. Moreover, there is an important association with p53 mutation, mammary cell dedifferentiation, and the concomitant acquisition of stemlike properties [62]. In particular, basal-like subtype shows high p53 mutation and *PROM1* up-regulation, which improve tumour cells aggressiveness [63] due to activation of angiogenesis and metastasis [64]; besides chemoresistance [65].
ILMN_1753101	VTCN1		-				V-set domain containing T cell activation inhibitor 1 (*VTCN1*; also known as *B7h.5, B7H4, B7-H4, B7S1, B7X, FLJ22418, Immune costimulatory protein B7-H4, PRO1291, Protein B7S1, T-cell costimulatory molecule B7x, UNQ659/PRO1291, VCTN1, V-set domain-containing T-cell activation inhibitor 1*) is found on the surface of antigen-presenting cells and interacts with ligands attached to receptors on the surface of T cells. The protein negatively regulates the immune response of T cells by reducing the production of cytokines and controlling the cell cycle progression [66,67]. High levels of the *VTCN1* mRNA and the related protein are associated with a number of cancers, including ovarian and breast cancers [68]. The up-regulation is also associated with tumour progression and linked to poor prognosis [69]. Although *VTCN1* detection is observed in PR-negative and HER2-negative tumours, the expression is independent of grade, stage, or other clinicopathologic variables [70].
ILMN_1798108	C6orf211		+				The chromosome 6 open reading frame 211 (C6orf211) is mapped in a region close to *ESR1* and other genes (*AKAP12* and *CCDC170*), suggesting further investigation of a possible connection with the *ESR1* transcription and the luminal subtype in breast cancer disease.

**Illumina Probe_ID**	**Symbol**	**Luminal A**	**Luminal B**	**Her2 enriched**	**Normal like**	**Basal like**	**Gene and Protein Review**
ILMN_1747911	CDK1		+				The cyclin-dependent kinase 1 (*CDK1*; also known as *CDC2, CDC28A, Cell division control protein 2 homolog, Cell division protein kinase 1, Cyclin-dependent kinase 1, DKFZp686L20222, MGC111195, P34CDC2, p34 protein kinase*) plays a key role in the control of the cell cycle by modulating the centrosome as well as mitotic onset; promotes G2-M transition, and regulates G1 progress and G1-S transition associated with multiple interphase cyclins. The kinase activity of this protein is controlled by cyclin accumulation and destruction through the cell cycle. In addition, CDK1 complexes phosphorylate several substrates that trigger centrosome separation, Golgi dynamics, nuclear envelope breakdown, chromosome condensation, and apoptosis. An abnormal phosphorylation occurs in cancer cell lines, as well as in primary breast tissues and lymphocytes. Moreover, high *CDK1* activity was linked to the absence of a full DNA damage response in mitotic cells [71]. Although the impact of this gene in breast cancers remains controversial, there is a significant association with unfavourable clinicopathologic feature such as high histologic grade, large tumour size, lymph node metastases and PR-negative tumours [72]. Ultimately, *CDK1* may be used as a predictive factor to identify patient's response to neoadjuvant chemotherapy [73-75].
ILMN_1666305	CDKN3		+				The protein encoded by cyclin-dependent kinase inhibitor 3 (*CDKN3*; also known as *CDI1, CDK2-associated dual-specificity phosphatase, CIP2, Cyclin-dependent kinase inhibitor 3, Cyclin-dependent kinase-interacting protein 2, Cyclin-dependent kinase interactor 1, FLJ25787, KAP, KAP1, Kinase-associated phosphatase, MGC70625*) belongs to the dual specificity protein phosphatase family, active toward substrates containing either phosphotyrosine or phosphoserine residues. CDKN3 is a cyclin-dependent kinase inhibitor, and interacts / dephosphorylates CDK2 kinase, thereby reducing its ability to phosphorylate the retinoblastoma protein (RB). Non-phosphorylated RB binds transcription factor E2F1 and prevents the G1-S transition. *CDKN3* was reported to be deleted, mutated, or overexpressed in several types of cancers, including breast tumours [76].
ILMN_1678535	ESR1		+	-		-	Estrogen receptor 1 (*ESR1*; also known as *DKFZp686N23123, ER, Era, ER-alpha, ESR, ESRA, Estradiol receptor, Estrogen receptor, NR3A1, Nuclear receptor subfamily 3 group A member 1*) encodes a protein receptor, a ligand-activated transcription factor composed of several domains important for hormone binding, DNA binding, and activation of transcription. Oestrogen and its receptors are central regulators of breast cancer disease and are associated with response to endocrine therapy. Down-regulation of *ESR1*, or eventual mutations, may indicate intrinsic resistance to tamoxifen, increased risk of tumour recurrence, and worse prognosis [77-79]; even though the mechanisms by which oestrogen receptor dictates tumour status are poorly understood [80].
ILMN_2149164	SFRP1		-		+		Major gene expression changes occur during progression of neoplastic cells, including down regulation of secreted frizzled-related protein 1 (*SFRP1*; also known as *FRP, FRP1, FRP-1, FrzA, SARP2, SARP-2, Secreted apoptosis-related protein 2, Secreted frizzled-related protein 1, sFRP-1*) [81]. SFRP1 functions as a negative regulator of Wnt/β-catenin pathway, implicated in several human cancers, including breast tumours and respective cell lines [82-89]. Reduced levels of *SFRP1* results in hyperplastic lesions and its loss may be a critical event in cancer initiation [90]. In breast carcinomas, *SFRP1* showed significant differences in methylation patterns between ER-negative and ER-positive tumours [91]. The hypermethylation of the *SFRP1* promoter and gene down-regulation has been widely reported in breast cancer [81,92-94] and associated with tumour invasion and decreased survival [95-98]. A potential combinatorial treatment - romidepsin and decitabine - has recently been administered in cell lines, promoting *SFRP1* reexpression with consequently proliferation inhibition and cell death induction via apoptosis [92].

Illumina Probe_ID	Symbol	Luminal A	Luminal B	Her2 enriched	Normal like	Basal like	Gene and Protein Review
ILMN_1788874	SERPINA3			-			Serpin peptidase inhibitor, clade A (alpha-1 antiproteinase, antitrypsin), member 3 (*SERPINA3*; also known as *AACT, ACT, alpha-1-antichymotrypsin, Alpha-1-antichymotrypsin, Cell growth-inhibiting gene 24/25 protein, GIG24, GIG25, MGC88254, Serpin A3*) encodes a plasma protease inhibitor and member of the serine protease inhibitor class. *SERPINA3* regulates the activity of neutrophil cathepsin G and is an oestrogen-induced gene. In breast cancer, the mRNA increased expression was reported as an indicator of good prognosis in oestrogen receptor positive breast cancer [99-101]. It is a maker of oestrogen regulation [102]; besides a predictor of tumour response to neoadjuvant chemotherapy [99].
ILMN_1785570	SUSD3*			-			The sushi domain containing 3 (*SUSD3*; also known as *MGC26847, Sushi domain-containing protein 3, UNQ9387/PRO34275*) down-regulated in breast carcinomas is associated with a malignant phenotype (short-term overall survival, endocrine insensitivity, triple-negative status, poor tumour differentiation). *SUSD3* is highly expressed in ER-positive breast tumours and the treatment with oestradiol may increase the gene expression in cancer cells [103]. The *SUSD3* abnormal mRNA levels and the protein function, however, are still unclear and require urgent investigation [100].
ILMN_1803236	CLCA2			+			The protein encoded by chloride channel accessory 2 (*CLCA2*; also known as *CACC, CACC3, CaCC-3, Calcium-activated chloride channel family member 2, Calcium-activated chloride channel protein 3, Calcium-activated chloride channel regulator 2, CLCRG2, FLJ97885, hCaCC-3, hCLCA2*) belongs to the calcium sensitive chloride conductance protein family. The protein plays a role in modulating chloride current across the plasma membrane in a calcium-dependent mode. CLCA2 is also involved in basal cell adhesion and/or stratification of squamous epithelia. In addition, the molecule is involved in the p53 network and may act as a tumour suppressor in breast and colorectal cancer, inhibiting cancer cell migration and invasion [104-106]. The mechanisms behind the silencing of *CLCA2* in luminal breast cancers and the up-regulation in HER2-enriched and basal-like subtypes, however, have not been elucidated. Ultimately, cell lines *CLCA2*-negative treated with demethylating agents restored the expression of the gene, suggesting an epigenetic control [107].
ILMN_2161820	GLYATL2*			+			The enzyme encoded by glycine-N-acyltransferase-like 2 (*GLYATL2*; also known as *Acyl-CoA:glycine N-acyltransferase-like protein 2, BXMAS2-10, GATF-B, Glycine N-acyltransferase-like protein 2, MGC24009*) conjugates medium- and long-chain saturated and unsaturated acyl-CoA esters to glycine, resulting in the production of N-oleoyl glycine and also N-arachidonoyl glycine. N-Oleoyl glycine and N-arachidonoyl glycine are identified as signalling molecules that regulate the perception of pain and body temperature, and also have anti-inflammatory properties [108]. *GLYATL2* is up-regulated in salivary gland and trachea, and detected also in spinal cord and skin fibroblasts. In addition, the high levels of the gene in skin and lung may indicate a role in barrier function/immune response and lipid signalling [109].
ILMN_1810978	MUCL1*			+			The mucin-like 1 (*MUCL1*; also known as *Mucin-like protein 1, Protein BS106, SBEM, Small breast epithelial mucin, UNQ590/PRO1160*) encodes a 90 amino acids glycoprotein that exhibits characteristics of members of the mucin family. The presence of a hydrophobic signal peptide within the protein sequence suggests that MUCL1 is a secreted and subjected to proteolytic processing. The putative gene is expressed only in mammary and salivary glands and it is promising as a new biomarker with high tissue specificity [110], besides with a great potential for predicting metastasis and response to neoadjuvant chemotherapy [111]. In breast cancer, the protein is more frequently observed in ER-negative than in ER-positive cancers, and positively associated with *HER2* overexpression. The evaluation of MUCL1 expression, nonetheless, needs to consider also the heterogeneity and different molecular subtypes [112]. In general, increased expression levels of *MUCL1* are strongly associated with higher tumour grades, lymph node metastasis [113] and reduced patient overall survival [114,115].

Illumina Probe_ID	Symbol	Luminal A	Luminal B	Her2 enriched	Normal like	Basal like	Gene and Protein Review
ILMN_1773459	SOX11*			+			SRY (sex determining region Y)-box 11 (*SOX11*; also known as *Transcription factor SOX-11*) encodes a protein involved in the regulation of embryonic development and in the determination of the cell fate. The protein acts as a transcriptional regulator after modelling a complex with other proteins. SOX11 functions in the developing nervous system and play a role in tumorigenesis. In breast cancer patients, the gene up-regulation might contribute to a proliferative genotype which may be linked to poor prognosis and therefore worse overall survival [116]. Interestingly, *SOX11* levels were higher in basal-like and HER2-enriched breast cancers compared with other subtypes [117].
ILMN_1674533	TRPV6			+			Transient receptor potential cation channel, subfamily V, member 6 (*TRPV6*; also known as *ABP/ZF, Calcium transport protein 1, CaT1, CAT1, CATL, CaT-L, CaT-like, ECaC2, ECAC2, Epithelial calcium channel 2, HSA277909, LP6728, Transient receptor potential cation channel subfamily V member 6, TrpV6, ZFAB*) encodes a member of a family of multipass membrane proteins that functions as calcium channels. The calcium selective cation channel is involved in Ca2+ uptake in various tissues, including Ca2+ reabsorption in intestine. The up-regulation of *TRPV6* at both the mRNA and protein levels is observed in several tumours such as breast, prostate, colon, thyroid and ovary; and in various tumour cell lines including those of colon, human leukaemia and prostate [118,119]. In particular, increased TRPV6 expression is a feature of ER-negative breast tumours (HER2-enriched and basal-like subtypes) and has been associated to patient decreased survival [120]. The exact mechanism underlying the *TRPV6*-mediated regulation of cancer progression and its downstream signalling, however, remain poorly understood [121]. Inhibitors of the TRPV6 channel have been investigated as potential targets for diagnosis, prognosis and/or therapeutic approaches in human cancers [122,123].
ILMN_1687235	HPN				-		The gene hepsin (*HPN*; also known as *Serine protease hepsin, TMPRSS1, Transmembrane protease serine 1*) encodes a type II transmembrane serine protease that is involved in diverse cellular functions, including cell growth and maintenance of cell morphology. The protein is cleaved into a catalytic serine protease chain and a non-catalytic scavenger receptor. The expression of the encoded protein is associated with the progression of several types of malignancies, nevertheless little is known about its clinical and biological significance in breast cancer. *HPN* is up-regulated in breast tumours; besides significantly associated with tumour stage, lymph node metastasis, oestrogen receptor positivity, and progesterone receptor positivity [124].
ILMN_1655915	MMP11				-		The matrix metallopeptidase 11 (stromelysin 3) (*MMP11*; also known as *Matrix metalloproteinase-11, MMP-11, SL-3, ST3, STMY3, Stromelysin-3*) is a member of the matrix metalloproteinase (MMP) family of proteases. These proteins are constituent of the extracellular matrix and act on the epithelial/connective interface in embryogenesis, wound healing, tissue involution, and reproduction. *MMP11* expressed in fibroblasts near areas of invasive carcinoma lead to the gain of metastatic potential for spread of tumour cells, with patterns of subsequent invasion and migration for different types of solid tumours and cell lines [125-129]. In breast cancer, higher expression level of *MMP11* is correlated with patients having poorly differentiated tumours, increased invasiveness, node metastasis, and worse prognosis [130-134]. *MMP11* gene expression analysis may also be used in clinical applications for breast cancer diagnosis, management and therapy [135,136]. Ultimately, the up-regulation of this gene is linked to other markers such as p53, ER and *HER2* [137].
ILMN_1711470	UBE2T				-		The ubiquitin-conjugating enzyme E2T (putative) (*UBE2T*; also known as *Cell proliferation-inducing gene 50 protein, HSPC150, PIG50, Ubiquitin carrier protein T, Ubiquitin-conjugating enzyme E2 T, Ubiquitin-protein ligase T*) accepts ubiquitin from the E1 complex and catalyses its covalent attachment to other proteins. The covalent conjugation of ubiquitin to proteins regulates diverse cellular pathways and proteins. Ubiquitin is transferred to a target protein through a concerted action of ubiquitin-activating enzyme (E1), ubiquitin-conjugating enzyme (E2), and ubiquitin ligase (E3). UBE2T acts as a specific E2 ubiquitin-conjugating enzyme for the Fanconi anemia complex and contribute to ubiquitination and degradation of BRCA1. The enzyme is up-regulated in different types of cancer including breast, bladder, lung, and prostate cancers; playing essential role in cell proliferation. In breast tumours, the gene up-regulation cause the decrease of the BRCA1 levels, however, major pathways involving UBE2T are still poorly understood [138].

Illumina Probe_ID	Symbol	Luminal A	Luminal B	Her2 enriched	Normal like	Basal like	Gene and Protein Review
ILMN_1789507	COL11A1				-		The collagen, type XI, alpha 1 (COL11A1; also known as *CO11A1, COLL6, Collagen alpha-1(XI) chain, STL2*) encodes one of the two alpha chains of type XI collagen, a minor fibrillar collagen. Type XI collagen is a heterotrimer and play an important role in fibrillogenesis by controlling lateral growth of collagen fibrils. *COL11A1* is expressed by both the epithelial and stromal compartments and its expression is deregulated in a range of cancers, such as breast and colon. In particular, molecules related to extracellular matrix remodelling (e.g. COL11A1) are differentially expressed in breast tumours 'in situ' and invasive; enriched in metastatic tumour cells [81,139,140].
ILMN_1740609	CCL15				+		The chemokine (C-C motif) ligand 15 (*CCL15*; also known as *C-C motif chemokine 15, Chemokine CC-2, HCC-2, HMRP-2B, Leukotactin-1, LKN1, Lkn-1, LKN-1, Macrophage inflammatory protein 5, MIP-1d, MIP-1D, MIP-1 delta, MIP5, MIP-5, Mrp-2b, MRP-2B, NCC3, NCC-3, SCYA15, SCYL3, Small-inducible cytokine A15, SY15*) encodes a secreted protein characterized by two adjacent cysteines, further processed into numerous smaller functional peptides. The protein has chemotactic factor that attracts T cells and monocytes; acts through C-C chemokine receptor type 1 (CCR1) and also binds to type 3 (CCR3). In hepatocellular carcinoma, the up-regulation of *CCL15* promotes cell migration and invasion [141]. High levels of *CCL15* also increase the expression of matrix metalloproteinase and induce angiogenesis [142].
ILMN_1651282	COL17A1*				+		The collagen, type XVII, alpha 1 (*COL17A1*; also known as *180 kDa bullous pemphigoid antigen 2, BA16H23.2, BP180, BPAG2, Bullous pemphigoid antigen 2, Collagen alpha-1(XVII) chain, FLJ60881, KIAA0204, LAD-1*) encodes the alpha chain of type XVII collagen, a transmembrane protein or a soluble form generated by proteolytic processing of the full length form. The protein is a structural component of hemidesmosomes, multiprotein complexes at the dermal-epidermal basement membrane zone that mediate adhesion of basal keratinocytes to the underlying membrane. Hemidesmosomal components are also implicated in signal transduction and thereby are able to influence cell growth, motility and differentiation. In neoplastic tissue, *COL17A1* aberrant expression depends on the stage of the tumour, down-regulated in mild dysplasia and up-regulation as the tumour further evolves [143].
ILMN_1723684	DARC				+		Duffy blood group, atypical chemokine receptor (*DARC*; also known as *CCBP1, CD234, Dfy, Duffy antigen/chemokine receptor, FY, Fy glycoprotein, Glycoprotein D, GPD, GpFy, Plasmodium vivax receptor, WBCQ1*) encodes a glycosylated membrane protein and a non-specific receptor for several chemokines. Polymorphisms in this gene are the basis of the Duffy blood group system. It is reported that DARC plays a negative regulatory role in human breast cancer. Overexpression of DARC protein in breast cancer cells leads to significant inhibition of tumorigenesis and metastasis [144-146]. DARC is also correlated with breast cancer incidence, axillary lymph node metastasis and overall survival [147].
ILMN_1809099	IL33*				+		Interleukin 33 (*IL33*; also known as *C9orf26, DKFZp586H0523, DVS27, IL1F11, IL-1F11, IL-33, Interleukin-1 family member 11, Interleukin-33, NFEHEV, NFHEV, NF-HEV, Nuclear factor from high endothelial venules, RP11-575C20.2*) is a member of the IL1 family that induces production of T helper-2 (Th2) associated cytokines. The protein acts as a chemoattractant tor Th2 cells, and amplifies immune responses during tissue injury. IL33 also functions as a chromatin-associated nuclear factor with transcriptional repressor properties. In breast cancer cells, a frequent overexpression is observed, though the gene deregulation is not clearly understood in the disease [148].

Illumina Probe_ID	Symbol	Luminal A	Luminal B	Her2 enriched	Normal like	Basal like	Gene and Protein Review
ILMN_1766650	FOXA1					-	The forkhead box A1 (*FOXA1*; also known as *Forkhead box protein A1, Hepatocyte nuclear factor 3-alpha, HNF3A, HNF-3A, HNF-3-alpha, MGC33105, TCF3A, TCF-3A, Transcription factor 3A*) encodes a member of the forkhead class of DNA-binding proteins. The nuclear factor is a transcriptional activator involved in embryonic development, establishment of tissue-specific gene expression and regulation of gene expression in differentiated tissues. The protein is also implicated in the development of multiple organs such as liver, pancreas, thyroid, prostate and breast. Basically, it modulates the transcriptional activity of nuclear hormone receptors [149]. FOXA1 acts in both androgen receptor (AR) and oestrogen receptor (ER), directing the binding location, and therefore the transcriptional activity [150-152]. In breast cancer, FOXA1 plays a pivotal role in mammary ductal morphogenesis [153], tumour early stage, drug response, and metastatic disease [154,155]. Mutation and SNP variation located in enhancer regions may alter FOXA1 binding affinity and affect breast cancer risk [156-159]. In addition, high expression of FOXA1 is correlated with luminal A subtype and it is a significant predictor of survival in patients with ER-positive tumours [149,160-162], and a marker of good prognosis [163-166]. Ultimately, genome analysis of ER-FOXA1 interactions is required to understand the molecular mechanisms of ER activity [167-170].
ILMN_1811387	TFF3					-	The trefoil factor 3 (intestinal) (*TFF3*; also known as *hITF, HITF, hP1.B, Intestinal trefoil factor, ITF, P1B, Polypeptide P1.B, TFI, Trefoil factor 3*) gene is translated in a stable secretory protein having at least one copy of the trefoil motif and a domain with three conserved disulphides. TFF3 functions as 'luminal epithelium guardian', involved in the maintenance and repair of the mucosa after damage. Besides, promotes the mobility of epithelial cells in healing processes. Up-regulation of *TFF3* is observed in various neoplastic diseases, including breast cancer, where the gene has been target as a biomarker [171]. TFF3 is induced by hormones such as oestrogen, and is usually combined with TFF1 expression in ER-positive malignant breast tumour cells [172,173]. Moreover, the *TFF3* levels are close to that of *ESR1*, yet reduce after tamoxifen treatment [32,174]. These genes are components of the 'luminal epithelial' signature defining a well-differentiated, low-grade intrinsic subtype of breast cancer: the luminal A [172]. Basal-like and claudin-low breast cancer subtypes showed frequent hypermethylation of the TFF3 promoter region [175,176].
ILMN_1738401	FOXC1					+	The forkhead box C1 (*FOXC1*; also known as *ARA, FKHL7, Forkhead box protein C1, Forkhead-related protein FKHL7, Forkhead-related transcription factor 3, FREAC3, FREAC-3, IGDA, IHG1, IRID1, RIEG3*) is part of the forkhead family of transcription factors, characterized by a common DNA-binding domain. All the mechanisms through *FOXC1* are not yet determined; however, the gene plays important roles in cell growth, survival, differentiation, and migration. *FOXC1* is identified as a functionally important biomarker of breast cancer aggressiveness, particularly associated with basal-like breast cancer subtype [177,178]. The gene up-regulation in breast tumour cells induces epithelial-mesenchymal transition, drug resistance, and increased cell proliferation and invasion [179].
ILMN_1689146	GABRP					+	Gamma-aminobutyric acid (GABA) A receptor, pi (*GABRP*; also known as *GABA(A) receptor subunit pi, Gamma-aminobutyric acid receptor subunit pi, MGC126386, MGC126387*) encodes a transmembrane protein composed by multisubunit in the chloride channel that mediates synaptic transmission in the central nervous system. The gene is also expressed in several non-neuronal tissues including the uterus, breast, and ovaries. In breast tissue, *GABRP* is mainly expressed in myoepithelial/basal cells, and the function is related to tissue contractility [172]. In breast cancer cells, the gene is normally up-regulated among ER-negative patients [180,181].

Illumina Probe_ID	Symbol	Luminal A	Luminal B	Her2 enriched	Normal like	Basal like	Gene and Protein Review
ILMN_1807423	IGF2BP3*					+	The protein encoded by insulin-like growth factor 2 mRNA binding protein 3 (*IGF2BP3*; also known as *CT98, DKFZp686F1078, hKOC, IGF2 mRNA-binding protein 3, IGF-II mRNA-binding protein 3, IMP3, IMP-3, Insulin-like growth factor 2 mRNA-binding protein 3, KH domain-containing protein overexpressed in cancer, KOC1, VICKZ3, VICKZ family member 3*) is primarily located in the nucleolus and belongs to a conserved family of RNA-binding proteins. The RNA-binding factor recruits target transcripts to cytoplasmic protein-RNA complexes (mRNPs), modulating the rate and location of endonuclease attacks or microRNA-mediated degradation. The protein, nonetheless, is involved not only in RNA synthesis and metabolism, but in various important aspects of cell function, such as cell polarization, migration, morphology, metabolism, proliferation and differentiation. IGF2BP3 is largely absent in adult tissues but *de novo* synthesized or severely up-regulated in various tumours and tumour-derived cells. In breast cancer, the up-regulation enhances tumour growth, angiogenesis and metastasis, resulting in poorer survival [182-184], and chemoresistance [185]. High expression has also been associated with triple-negative breast carcinomas [186-188].
ILMN_1692938	PSAT1					+	The phosphoserine aminotransferase 1 (*PSAT1*; also known as *EPIP, MGC1460, Phosphohydroxythreonine aminotransferase, Phosphoserine aminotransferase, PSA, PSAT*) encodes a member of the class-V pyridoxal-phosphate-dependent aminotransferase family. Mutations in this gene are associated with phosphoserine aminotransferase deficiency. *PSAT1* methylation and aberrant expression are strongly correlated with specific clinical and pathologic features of breast cancer. Notably, the *PSAT1* hypermethylation is associated with low-grade, low-proliferation, hormone receptor ER-positive, lymph node positive breast cancer in post-menopausal women [189]; besides it is an indicator of response to tamoxifen endocrine therapy [190]. On the other hand, high expression of *PSAT1* is associated with decreased relapse-free and overall survival of patients, and linked to malignant phenotypic features of breast cancer [189].
ILMN_1668766	ROPN1					+	The rhophilin associated tail protein 1 (*ROPN1*; also known as *Cancer/testis antigen 91, CT91, DKFZp434B1222, ODF6, Rhophilin-associated protein 1A, RHPNAP1, ROPN1A, ropporin, Ropporin-1A*) is an important reproduction related gene. The protein is involved in sperm maturation, motility, capacitation, hyperactivation and acrosome reaction. Other important functions such as cAMP-dependent protein kinase regulator activity, protein binding activity, phosphorylation and signal transduction regulation were reported; even though ROPN1 requires further investigation [191]. Recently, *ROPN1* was validated with diagnostic significance in basal-like breast cancer cells as one of the conserved elements of the *SOX10* signature [192].

*Elements named according to Human HT (Illumina HT-12 v3) and matching different regions of the genome with more than one annotation in UCSC and iHOP.
Note: Genes are described according to the RefSeq (Reference Sequence) and UniProt databases; and improved with related citations in PubMed.

References

1. Ciriello G, Sinha R, Hoadley KA, Jacobsen AS, Reva B, et al. (2013) The molecular diversity of Luminal A breast tumors. Breast cancer research and treatment 141: 409-420.
2. Hegyi K, Egervari K, Sandor Z, Mehes G (2012) Aurora kinase B expression in breast carcinoma: cell kinetic and genetic aspects. Pathobiology 79: 314-322.
3. Ahn SG, Lee HM, Lee HW, Lee SA, Lee SR, et al. (2013) Prognostic discrimination using a 70-gene signature among patients with estrogen receptor-positive breast cancer and an intermediate 21-gene recurrence score. International journal of molecular sciences 14: 23685-23699.
4. Fiskus W, Hembruff SL, Rao R, Sharma P, Balusu R, et al. (2012) Co-treatment with vorinostat synergistically enhances activity of Aurora kinase inhibitor against human breast cancer cells. Breast cancer research and treatment 135: 433-444.
5. Gully CP, Zhang F, Chen J, Yeung JA, Velazquez-Torres G, et al. (2010) Antineoplastic effects of an Aurora B kinase inhibitor in breast cancer. Molecular cancer 9: 42.
6. Arbitrario JP, Belmont BJ, Evanchik MJ, Flanagan WM, Fucini RV, et al. (2010) SNS-314, a pan-Aurora kinase inhibitor, shows potent anti-tumor activity and dosing flexibility in vivo. Cancer chemotherapy and pharmacology 65: 707-717.
7. Hardwicke MA, Oleykowski CA, Plant R, Wang J, Liao Q, et al. (2009) GSK1070916, a potent Aurora B/C kinase inhibitor with broad antitumor activity in tissue culture cells and human tumor xenograft models. Molecular cancer therapeutics 8: 1808-1817.
8. Ueki T, Nishidate T, Park JH, Lin ML, Shimo A, et al. (2008) Involvement of elevated expression of multiple cell-cycle regulator, DTL/RAMP (denticleless/RA-regulated nuclear matrix associated protein), in the growth of breast cancer cells. Oncogene 27: 5672-5683.
9. Kalous O, Conklin D, Desai AJ, Dering J, Goldstein J, et al. (2013) AMG 900, pan-Aurora kinase inhibitor, preferentially inhibits the proliferation of breast cancer cell lines with dysfunctional p53. Breast cancer research and treatment 141: 397-408.
10. Bush TL, Payton M, Heller S, Chung G, Hanestad K, et al. (2013) AMG 900, a small-molecule inhibitor of aurora kinases, potentiates the activity of microtubule-targeting agents in human metastatic breast cancer models. Molecular cancer therapeutics 12: 2356-2366.
11. Romanelli A, Clark A, Assayag F, Chateau-Joubert S, Poupon MF, et al. (2012) Inhibiting aurora kinases reduces tumor growth and suppresses tumor recurrence after chemotherapy in patient-derived triple-negative breast cancer xenografts. Molecular cancer therapeutics 11: 2693-2703.
12. Sanchez-Bailon MP, Calcabrini A, Gomez-Dominguez D, Morte B, Martin-Forero E, et al. (2012) Src kinases catalytic activity regulates proliferation, migration and invasiveness of MDA-MB-231 breast cancer cells. Cellular signalling 24: 1276-1286.
13. Soncini C, Carpinelli P, Gianellini L, Fancelli D, Vianello P, et al. (2006) PHA-680632, a novel Aurora kinase inhibitor with potent antitumoral activity. Clinical cancer research 12: 4080-4089.
14. Mertsch S, Schmitz N, Jeibmann A, Geng JG, Paulus W, et al. (2008) Slit2 involvement in glioma cell migration is mediated by Robo1 receptor. Journal of neuro-oncology 87: 1-7.
15. Zhang W, Kawanishi M, Miyake K, Kagawa M, Kawai N, et al. (2010) Association between YKL-40 and adult primary astrocytoma. Cancer 116: 2688-2697.

16. Carretero M, Ruiz-Torres M, Rodriguez-Corsino M, Barthelemy I, Losada A (2013) Pds5B is required for cohesion establishment and Aurora B accumulation at centromeres. The EMBO journal 32: 2938-2949.
17. Nguyen MH, Koinuma J, Ueda K, Ito T, Tsuchiya E, et al. (2010) Phosphorylation and activation of cell division cycle associated 5 by mitogen-activated protein kinase play a crucial role in human lung carcinogenesis. Cancer Research 70: 5337-5347.
18. Murphy KJ, Ter Horst JP, Cassidy AW, DeSouza IE, Morgunova M, et al. (2010) Temporal dysregulation of cortical gene expression in the isolation reared Wistar rat. Journal of neurochemistry 113: 601-614.
19. Inoda S, Hirohashi Y, Torigoe T, Nakatsugawa M, Kiriyama K, et al. (2009) Cep55/c10orf3, a tumor antigen derived from a centrosome residing protein in breast carcinoma. Journal of immunotherapy 32: 474-485.
20. Colak D, Nofal A, Albakheet A, Nirmal M, Jeprel H, et al. (2013) Age-specific gene expression signatures for breast tumors and cross-species conserved potential cancer progression markers in young women. PLoS One 8: e63204.
21. Martin KJ, Patrick DR, Bissell MJ, Fournier MV (2008) Prognostic breast cancer signature identified from 3D culture model accurately predicts clinical outcome across independent datasets. PLoS One 3: e2994.
22. Lin ML, Park JH, Nishidate T, Nakamura Y, Katagiri T (2007) Involvement of maternal embryonic leucine zipper kinase (MELK) in mammary carcinogenesis through interaction with Bcl-G, a pro-apoptotic member of the Bcl-2 family. Breast cancer research 9: R17.
23. Agnati LF, Genedani S, Leo G, Forni A, Woods AS, et al. (2007) Abeta peptides as one of the crucial volume transmission signals in the trophic units and their interactions with homocysteine. Physiological implications and relevance for Alzheimer's disease. Journal of neural transmission 114: 21-31.
24. Canevari G, Re Depaolini S, Cucchi U, Bertrand JA, Casale E, et al. (2013) Structural insight into maternal embryonic leucine zipper kinase (MELK) conformation and inhibition toward structure-based drug design. Biochemistry 52: 6380-6387.
25. Mahasenan KV, Li C (2012) Novel inhibitor discovery through virtual screening against multiple protein conformations generated via ligand-directed modeling: a maternal embryonic leucine zipper kinase example. Journal of chemical information and modeling 52: 1345-1355.
26. Hebbard LW, Maurer J, Miller A, Lesperance J, Hassell J, et al. (2010) Maternal embryonic leucine zipper kinase is upregulated and required in mammary tumor-initiating cells in vivo. Cancer Research 70: 8863-8873.
27. Pickard MR, Green AR, Ellis IO, Caldas C, Hedge VL, et al. (2009) Dysregulated expression of Fau and MELK is associated with poor prognosis in breast cancer. Breast Cancer Research 11: R60.
28. Warsow G, Struckmann S, Kerkhoff C, Reimer T, Engel N, et al. (2013) Differential network analysis applied to preoperative breast cancer chemotherapy response. PLoS One 8: e81784.
29. Loussouarn D, Campion L, Leclair F, Campone M, Charbonnel C, et al. (2009) Validation of UBE2C protein as a prognostic marker in node-positive breast cancer. British journal of cancer 101: 166-173.
30. Rawat A, Gopal G, Selvaluxmy G, Rajkumar T (2013) Inhibition of ubiquitin conjugating enzyme UBE2C reduces proliferation and sensitizes breast cancer cells to radiation, doxorubicin, tamoxifen and letrozole. Cellular oncology 36: 459-467.
31. Psyrri A, Kalogeras KT, Kronenwett R, Wirtz RM, Batistatou A, et al. (2012) Prognostic significance of UBE2C mRNA expression in high-risk early breast cancer. A Hellenic Cooperative Oncology Group (HeCOG) Study. Annals of oncology 23: 1422-1427.

32. Taylor KJ, Sims AH, Liang L, Faratian D, Muir M, et al. (2010) Dynamic changes in gene expression in vivo predict prognosis of tamoxifen-treated patients with breast cancer. Breast Cancer Research 12: R39.
33. Parris TZ, Kovacs A, Aziz L, Hajizadeh S, Nemes S, et al. (2014) Additive effect of the AZGP1, PIP, S100A8 and UBE2C molecular biomarkers improves outcome prediction in breast carcinoma. International journal of cancer 134: 1617-1629.
34. Jager D, Filonenko V, Gout I, Frosina D, Eastlake-Wade S, et al. (2007) NY-BR-1 is a Differentiation Antigen of the Mammary Gland. Applied Immunohistochemistry & Molecular Morphology 15: 77-83.
35. Woodard AH, Yu J, Dabbs DJ, Beriwal S, Florea AV, et al. (2011) NY-BR-1 and PAX8 immunoreactivity in breast, gynecologic tract, and other CK7+ carcinomas: potential use for determining site of origin. American journal of clinical pathology 136: 428-435.
36. Varga Z, Theurillat JP, Filonenko V, Sasse B, Odermatt B, et al. (2006) Preferential nuclear and cytoplasmic NY-BR-1 protein expression in primary breast cancer and lymph node metastases. Clinical cancer research 12: 2745-2751.
37. Seil I, Frei C, Sultmann H, Knauer SK, Engels K, et al. (2007) The differentiation antigen NY-BR-1 is a potential target for antibody-based therapies in breast cancer. International journal of cancer 120: 2635-2642.
38. Balafoutas D, zur Hausen A, Mayer S, Hirschfeld M, Jaeger M, et al. (2013) Cancer testis antigens and NY-BR-1 expression in primary breast cancer: prognostic and therapeutic implications. BMC cancer 13: 271.
39. Giger O, Caduff R, O'Meara A, Diener PA, Knuth A, et al. (2010) Frequent expression of the breast differentiation antigen NY-BR-1 in mammary and extramammary Paget's disease. Pathology international 60: 726-734.
40. Theurillat JP, Zurrer-Hardi U, Varga Z, Storz M, Probst-Hensch NM, et al. (2007) NY-BR-1 protein expression in breast carcinoma: a mammary gland differentiation antigen as target for cancer immunotherapy. Cancer immunology, immunotherapy 56: 1723-1731.
41. Theurillat JP, Zurrer-Hardi U, Varga Z, Barghorn A, Saller E, et al. (2008) Distinct expression patterns of the immunogenic differentiation antigen NY-BR-1 in normal breast, testis and their malignant counterparts. International journal of cancer 122: 1585-1591.
42. Ikeda H, Taira N, Hara F, Fujita T, Yamamoto H, et al. (2010) The estrogen receptor influences microtubule-associated protein tau (MAPT) expression and the selective estrogen receptor inhibitor fulvestrant downregulates MAPT and increases the sensitivity to taxane in breast cancer cells. Breast Cancer Research 12: R43.
43. Valet F, de Cremoux P, Spyratos F, Servant N, Dujaric ME, et al. (2013) Challenging single- and multi-probesets gene expression signatures of pathological complete response to neoadjuvant chemotherapy in breast cancer: experience of the REMAGUS 02 phase II trial. Breast 22: 1052-1059.
44. Kotoula V, Kalogeras KT, Kouvatseas G, Televantou D, Kronenwett R, et al. (2013) Sample parameters affecting the clinical relevance of RNA biomarkers in translational breast cancer research. Virchows Arch 462: 141-154.
45. Spicakova T, O'Brien MM, Duran GE, Sweet-Cordero A, Sikic BI (2010) Expression and silencing of the microtubule-associated protein Tau in breast cancer cells. Molecular cancer therapeutics 9: 2970-2981.
46. Tanaka S, Nohara T, Iwamoto M, Sumiyoshi K, Kimura K, et al. (2009) Tau expression and efficacy of paclitaxel treatment in metastatic breast cancer. Cancer chemotherapy and pharmacology 64: 341-346.

47. Fountzilas G, Kotoula V, Pectasides D, Kouvatseas G, Timotheadou E, et al. (2013) Ixabepilone administered weekly or every three weeks in HER2-negative metastatic breast cancer patients; a randomized non-comparative phase II trial. PloS one 8: e69256.
48. Mihaly Z, Kormos M, Lanczky A, Dank M, Budczies J, et al. (2013) A meta-analysis of gene expression-based biomarkers predicting outcome after tamoxifen treatment in breast cancer. Breast cancer research and treatment 140: 219-232.
49. Wang K, Deng QT, Liao N, Zhang GC, Liu YH, et al. (2013) Tau expression correlated with breast cancer sensitivity to taxanes-based neoadjuvant chemotherapy. Tumour biology 34: 33-38.
50. Fletcher GC, Patel S, Tyson K, Adam PJ, Schenker M, et al. (2003) hAG-2 and hAG-3, human homologues of genes involved in differentiation, are associated with oestrogen receptor-positive breast tumours and interact with metastasis gene C4.4a and dystroglycan. British journal of cancer 88: 579-585.
51. Persson S, Rosenquist M, Knoblach B, Khosravi-Far R, Sommarin M, et al. (2005) Diversity of the protein disulfide isomerase family: identification of breast tumor induced Hag2 and Hag3 as novel members of the protein family. Molecular phylogenetics and evolution 36: 734-740.
52. Kim SJ, Kang H, Chang HL, Jung YC, Sim H, et al. (2008) Promoter hypomethylation of the N-acetyltransferase 1 gene in breast cancer. Oncology Reports 19: 663-668.
53. Sim E, Walters K, Boukouvala S (2008) Arylamine N-acetyltransferases: from structure to function. Drug metabolism reviews 40: 479-510.
54. Sim E, Lack N, Wang CJ, Long H, Westwood I, et al. (2008) Arylamine N-acetyltransferases: structural and functional implications of polymorphisms. Toxicology 254: 170-183.
55. Kabbage M, Trimeche M, Ben Nasr H, Hammann P, Kuhn L, et al. (2012) Expression of the molecular chaperone alphaB-crystallin in infiltrating ductal breast carcinomas and the significance thereof: an immunohistochemical and proteomics-based strategy. Tumour biology 33: 2279-2288.
56. Ruan Q, Han S, Jiang WG, Boulton ME, Chen ZJ, et al. (2011) alphaB-crystallin, an effector of unfolded protein response, confers anti-VEGF resistance to breast cancer via maintenance of intracrine VEGF in endothelial cells. Molecular cancer research 9: 1632-1643.
57. Campbell-Lloyd AJ, Mundy J, Deva R, Lampe G, Hawley C, et al. (2013) Is alpha-B crystallin an independent marker for prognosis in lung cancer? Heart, lung & circulation 22: 759-766.
58. Cortesi L, Barchetti A, De Matteis E, Rossi E, Della Casa L, et al. (2009) Identification of protein clusters predictive of response to chemotherapy in breast cancer patients. Journal of proteome research 8: 4916-4933.
59. van de Rijn M, Perou CM, Tibshirani R, Haas P, Kallioniemi O, et al. (2002) Expression of Cytokeratins 17 and 5 Identifies a Group of Breast Carcinomas with Poor Clinical Outcome. American Journal of Pathology 161.
60. Gorski JJ, James CR, Quinn JE, Stewart GE, Staunton KC, et al. (2010) BRCA1 transcriptionally regulates genes associated with the basal-like phenotype in breast cancer. Breast cancer research and treatment 122: 721-731.
61. Liu Q, Li JG, Zheng XY, Jin F, Dong HT (2009) Expression of CD133, PAX2, ESA, and GPR30 in invasive ductal breast carcinomas. Chinese medical journal 122: 2763-2769.
62. Coradini D, Fornili M, Ambrogi F, Boracchi P, Biganzoli E (2012) TP53 mutation, epithelial-mesenchymal transition, and stemlike features in breast cancer subtypes. Journal of biomedicine & biotechnology 2012: 254085.

63. Bertheau P, Turpin E, Rickman DS, Espie M, de Reynies A, et al. (2007) Exquisite Sensitivity of TP53 Mutant and Basal Breast Cancers to a Dose-Dense Epirubicin・Cyclophosphamide Regimen. PLoS Medicine 4: 585-594.
64. Liu N, Yu Q, Liu TJ, Gebreamlak EP, Wang SL, et al. (2012) P-cadherin expression and basal-like subtype in breast cancers. Medical oncology 29: 2606-2612.
65. Nadal R, Ortega FG, Salido M, Lorente JA, Rodriguez-Rivera M, et al. (2013) CD133 expression in circulating tumor cells from breast cancer patients: potential role in resistance to chemotherapy. International journal of cancer 133: 2398-2407.
66. Qian Y, Shen L, Cheng L, Wu Z, Yao H (2011) B7-H4 expression in various tumors determined using a novel developed monoclonal antibody. Clinical and experimental medicine 11: 163-170.
67. Suh WK, Wang S, Duncan GS, Miyazaki Y, Cates E, et al. (2006) Generation and characterization of B7-H4/B7S1/B7x-deficient mice. Molecular and cellular biology 26: 6403-6411.
68. Salceda S, Tang T, Kmet M, Munteanu A, Ghosh M, et al. (2005) The immunomodulatory protein B7-H4 is overexpressed in breast and ovarian cancers and promotes epithelial cell transformation. Experimental cell research 306: 128-141.
69. Heinonen H, Nieminen A, Saarela M, Kallioniemi A, Klefstrom J, et al. (2008) Deciphering downstream gene targets of PI3K/mTOR/p70S6K pathway in breast cancer. BMC Genomics 9: 348.
70. Tringler B, Zhuo S, Pilkington G, Torkko KC, Singh M, et al. (2005) B7-h4 is highly expressed in ductal and lobular breast cancer. Clinical cancer research 11: 1842-1848.
71. Zhang W, Peng G, Lin SY, Zhang P (2011) DNA damage response is suppressed by the high cyclin-dependent kinase 1 activity in mitotic mammalian cells. The Journal of biological chemistry 286: 35899-35905.
72. Kim SJ, Nakayama S, Miyoshi Y, Taguchi T, Tamaki Y, et al. (2008) Determination of the specific activity of CDK1 and CDK2 as a novel prognostic indicator for early breast cancer. Annals of oncology 19: 68-72.
73. Kim SJ, Nakayama S, Shimazu K, Tamaki Y, Akazawa K, et al. (2012) Recurrence risk score based on the specific activity of CDK1 and CDK2 predicts response to neoadjuvant paclitaxel followed by 5-fluorouracil, epirubicin and cyclophosphamide in breast cancers. Annals of oncology 23: 891-897.
74. Torikoshi Y, Gohda K, Davis ML, Symmans WF, Pusztai L, et al. (2013) Novel functional assay for spindle-assembly checkpoint by cyclin-dependent kinase activity to predict taxane chemosensitivity in breast tumor patient. Journal of Cancer 4: 697-702.
75. Xia Q, Cai Y, Peng R, Wu G, Shi Y, et al. (2014) The CDK1 inhibitor RO3306 improves the response of BRCA-pro fi cient breast cancer cells to PARP inhibition. International journal of oncology 44: 735-744.
76. Yu YN, Yip GW, Tan PH, Thike AA, Matsumoto K, et al. (2010) Y-box binding protein 1 is up-regulated in proliferative breast cancer and its inhibition deregulates the cell cycle. International Journal of Oncology 37.
77. Kim C, Tang G, Pogue-Geile KL, Costantino JP, Baehner FL, et al. (2011) Estrogen receptor (ESR1) mRNA expression and benefit from tamoxifen in the treatment and prevention of estrogen receptor-positive breast cancer. Journal of clinical oncology 29: 4160-4167.
78. Stossi F, Madak-Erdogan Z, Katzenellenbogen BS (2012) Macrophage-elicited loss of estrogen receptor-alpha in breast cancer cells via involvement of MAPK and c-Jun at the ESR1 genomic locus. Oncogene 31: 1825-1834.

79. Aguilar H, Sole X, Bonifaci N, Serra-Musach J, Islam A, et al. (2010) Biological reprogramming in acquired resistance to endocrine therapy of breast cancer. Oncogene 29: 6071-6083.
80. Dunbier AK, Anderson H, Ghazoui Z, Lopez-Knowles E, Pancholi S, et al. (2011) ESR1 is co-expressed with closely adjacent uncharacterised genes spanning a breast cancer susceptibility locus at 6q25.1. PLoS genetics 7: e1001382.
81. Vargas AC, McCart Reed AE, Waddell N, Lane A, Reid LE, et al. (2012) Gene expression profiling of tumour epithelial and stromal compartments during breast cancer progression. Breast cancer research and treatment 135: 153-165.
82. Gauger KJ, Chenausky KL, Murray ME, Schneider SS (2011) SFRP1 reduction results in an increased sensitivity to TGF-beta signaling. BMC Cancer 11: 59.
83. Mukherjee N, Bhattacharya N, Alam N, Roy A, Roychoudhury S, et al. (2012) Subtype-specific alterations of the Wnt signaling pathway in breast cancer: clinical and prognostic significance. Cancer science 103: 210-220.
84. Gostner JM, Fong D, Wrulich OA, Lehne F, Zitt M, et al. (2011) Effects of EpCAM overexpression on human breast cancer cell lines. BMC Cancer 11: 45.
85. Matsuda Y, Schlange T, Oakeley EJ, Boulay A, Hynes NE (2009) WNT signaling enhances breast cancer cell motility and blockade of the WNT pathway by sFRP1 suppresses MDA-MB-231 xenograft growth. Breast Cancer Research 11: R32.
86. Suzuki H, Toyota M, Carraway H, Gabrielson E, Ohmura T, et al. (2008) Frequent epigenetic inactivation of Wnt antagonist genes in breast cancer. British journal of cancer 98: 1147-1156.
87. Dahl E, Wiesmann F, Woenckhaus M, Stoehr R, Wild PJ, et al. (2007) Frequent loss of SFRP1 expression in multiple human solid tumours: association with aberrant promoter methylation in renal cell carcinoma. Oncogene 26: 5680-5691.
88. Shulewitz M, Soloviev I, Wu T, Koeppen H, Polakis P, et al. (2006) Repressor roles for TCF-4 and Sfrp1 in Wnt signaling in breast cancer. Oncogene 25: 4361-4369.
89. Ugolini F, Charafe-Jauffret E, Bardou VJ, Geneix J, Adelaide J, et al. (2001) WNT pathway and mammary carcinogenesis: loss of expression of candidate tumor suppressor gene SFRP1 in most invasive carcinomas except of the medullary type. Oncogene 20: 5810-5817.
90. Dumont N, Crawford YG, Sigaroudinia M, Nagrani SS, Wilson MB, et al. (2009) Human mammary cancer progression model recapitulates methylation events associated with breast premalignancy. Breast Cancer Research 11: R87.
91. Park SY, Kwon HJ, Choi Y, Lee HE, Kim SW, et al. (2012) Distinct patterns of promoter CpG island methylation of breast cancer subtypes are associated with stem cell phenotypes. Modern pathology 25: 185-196.
92. Cooper SJ, von Roemeling CA, Kang KH, Marlow LA, Grebe SK, et al. (2012) Reexpression of tumor suppressor, sFRP1, leads to antitumor synergy of combined HDAC and methyltransferase inhibitors in chemoresistant cancers. Molecular cancer therapeutics 11: 2105-2115.
93. Browne EP, Punska EC, Lenington S, Otis CN, Anderton DL, et al. (2011) Increased promoter methylation in exfoliated breast epithelial cells in women with a previous breast biopsy. Epigenetics 6: 1425-1435.
94. Yang ZQ, Liu G, Bollig-Fischer A, Haddad R, Tarca AL, et al. (2009) Methylation-associated silencing of SFRP1 with an 8p11-12 amplification inhibits canonical and non-canonical WNT pathways in breast cancers. International journal of cancer 125: 1613-1621.

95. Martin-Manso G, Calzada MJ, Chuman Y, Sipes JM, Xavier CP, et al. (2011) sFRP-1 binds via its netrin-related motif to the N-module of thrombospondin-1 and blocks thrombospondin-1 stimulation of MDA-MB-231 breast carcinoma cell adhesion and migration. Archives of biochemistry and biophysics 509: 147-156.
96. Veeck J, Niederacher D, An H, Klopocki E, Wiesmann F, et al. (2006) Aberrant methylation of the Wnt antagonist SFRP1 in breast cancer is associated with unfavourable prognosis. Oncogene 25: 3479-3488.
97. Gauger KJ, Schneider SS (2014) Tumour supressor secreted frizzled related protein 1 regulates p53-mediated apoptosis. Cell biology international 38: 124-130.
98. Klopocki E, Kristiansen G, Wild PJ, Klaman I, Castanos-Velez E, et al. (2004) Loss of SFRP1 is associated with breast cancer progression and poor prognosis in early stage tumors. International journal of oncology 25: 641-649.
99. Sano H, Wada S, Eguchi H, Osaki A, Saeki T, et al. (2012) Quantitative prediction of tumor response to neoadjuvant chemotherapy in breast cancer: novel marker genes and prediction model using the expression levels. Breast Cancer 19: 37-45.
100. Cimino D, Fuso L, Sfiligoi C, Biglia N, Ponzone R, et al. (2008) Identification of new genes associated with breast cancer progression by gene expression analysis of predefined sets of neoplastic tissues. International journal of cancer 123: 1327-1338.
101. Yamamura J, Miyoshi Y, Tamaki Y, Taguchi T, Iwao K, et al. (2004) mRNA expression level of estrogen-inducible gene, alpha 1-antichymotrypsin, is a predictor of early tumor recurrence in patients with invasive breast cancers. Cancer science 95: 887-892.
102. Miller WR, Larionov A (2010) Changes in expression of oestrogen regulated and proliferation genes with neoadjuvant treatment highlight heterogeneity of clinical resistance to the aromatase inhibitor, letrozole. Breast Cancer Research 12: R52.
103. Moy I, Todorovic V, Dubash AD, Coon JS, Parker JB, et al. (2014) Estrogen-dependent sushi domain containing 3 regulates cytoskeleton organization and migration in breast cancer cells. Oncogene.
104. Sasaki Y, Koyama R, Maruyama R, Hirano T, Tamura M, et al. (2012) CLCA2, a target of the p53 family, negatively regulates cancer cell migration and invasion. Cancer biology & therapy 13: 1512-1521.
105. Walia V, Ding M, Kumar S, Nie D, Premkumar LS, et al. (2009) hCLCA2 Is a p53-Inducible Inhibitor of Breast Cancer Cell Proliferation. Cancer Research 69: 6624-6632.
106. Walia V, Yu Y, Cao D, Sun M, McLean JR, et al. (2012) Loss of breast epithelial marker hCLCA2 promotes epithelial-to-mesenchymal transition and indicates higher risk of metastasis. Oncogene 31: 2237-2246.
107. Li X, Cowell JK, Sossey-Alaoui K (2004) CLCA2 tumour suppressor gene in 1p31 is epigenetically regulated in breast cancer. Oncogene 23: 1474-1480.
108. Waluk DP, Sucharski F, Sipos L, Silberring J, Hunt MC (2012) Reversible lysine acetylation regulates activity of human glycine N-acyltransferase-like 2 (hGLYATL2): implications for production of glycine-conjugated signaling molecules. The Journal of biological chemistry 287: 16158-16167.
109. Waluk DP, Schultz N, Hunt MC (2010) Identification of glycine N-acyltransferase-like 2 (GLYATL2) as a transferase that produces N-acyl glycines in humans. FASEB Journal 24: 2795-2803.
110. Liu L, Liu Z, Qu S, Zheng Z, Liu Y, et al. (2013) Small breast epithelial mucin tumor tissue expression is associated with increased risk of recurrence and death in triple-negative breast cancer patients. Diagnostic pathology 8: 71.

111. Liu ZZ, Xie XD, Qu SX, Zheng ZD, Wang YK (2010) Small breast epithelial mucin (SBEM) has the potential to be a marker for predicting hematogenous micrometastasis and response to neoadjuvant chemotherapy in breast cancer. Clinical & experimental metastasis 27: 251-259.
112. Valladares-Ayerbes M, Iglesias-Diaz P, Diaz-Prado S, Ayude D, Medina V, et al. (2009) Diagnostic accuracy of small breast epithelial mucin mRNA as a marker for bone marrow micrometastasis in breast cancer: a pilot study. Journal of cancer research and clinical oncology 135: 1185-1195.
113. Weigelt B, Verduijn P, Bosma AJ, Rutgers EJ, Peterse HL, et al. (2004) Detection of metastases in sentinel lymph nodes of breast cancer patients by multiple mRNA markers. British journal of cancer 90: 1531-1537.
114. Skliris GP, Hube F, Gheorghiu I, Mutawe MM, Penner C, et al. (2008) Expression of small breast epithelial mucin (SBEM) protein in tissue microarrays (TMAs) of primary invasive breast cancers. Histopathology 52: 355-369.
115. Miksicek RJ, Myal Y, Watson PH, Walker C, Murphy LC, et al. (2002) Identification of a Novel Breast- and Salivary Gland-specific, Mucin-like Gene Strongly Expressed in Normal and Tumor Human Mammary Epithelium. Cancer Research 62: 2736-2740.
116. Lopez FJ, Cuadros M, Cano C, Concha A, Blanco A (2012) Biomedical application of fuzzy association rules for identifying breast cancer biomarkers. Medical & biological engineering & computing 50: 981-990.
117. Zvelebil M, Oliemuller E, Gao Q, Wansbury O, Mackay A, et al. (2013) Embryonic mammary signature subsets are activated in Brca1-/- and basal-like breast cancers. Breast Cancer Research 15: R25.
118. Bolanz KA, Hediger MA, Landowski CP (2008) The role of TRPV6 in breast carcinogenesis. Molecular cancer therapeutics 7: 271-279.
119. Bowen CV, DeBay D, Ewart HS, Gallant P, Gormley S, et al. (2013) In vivo detection of human TRPV6-rich tumors with anti-cancer peptides derived from soricidin. PLoS One 8: e58866.
120. Peters AA, Simpson PT, Bassett JJ, Lee JM, Da Silva L, et al. (2012) Calcium channel TRPV6 as a potential therapeutic target in estrogen receptor-negative breast cancer. Molecular cancer therapeutics 11: 2158-2168.
121. Kim SY, Yang D, Myeong J, Ha K, Kim SH, et al. (2013) Regulation of calcium influx and signaling pathway in cancer cells via TRPV6-Numb1 interaction. Cell Calcium 53: 102-111.
122. Landowski CP, Bolanz KA, Suzuki Y, Hediger MA (2011) Chemical inhibitors of the calcium entry channel TRPV6. Pharmaceutical research 28: 322-330.
123. Dhennin-Duthille I, Gautier M, Faouzi M, Guilbert A, Brevet M, et al. (2011) High expression of transient receptor potential channels in human breast cancer epithelial cells and tissues: correlation with pathological parameters. Cellular physiology and biochemistry 28: 813-822.
124. Xing P, Li J-g, Jin F, Zhao T-t, Liu Q, et al. (2011) Clinical and Biological Significance of Hepsin Overexpression in Breast Cancer. Journal of Investigative Medicine 59: 803-810.
125. Delassus GS, Cho H, Eliceiri GL (2011) New signaling pathways from cancer progression modulators to mRNA expression of matrix metalloproteinases in breast cancer cells. Journal of cellular physiology 226: 3378-3384.
126. Delassus GS, Cho H, Park J, Eliceiri GL (2008) New pathway links from cancer-progression determinants to gene expression of matrix metalloproteinases in breast cancer cells. Journal of cellular physiology 217: 739-744.
127. Kwon YJ, Hurst DR, Steg AD, Yuan K, Vaidya KS, et al. (2011) Gli1 enhances migration and invasion via up-regulation of MMP-11 and promotes metastasis in ERalpha negative breast cancer cell lines. Clinical & experimental metastasis 28: 437-449.

128. Min KW, Kim DH, Do SI, Pyo JS, Kim K, et al. (2013) Diagnostic and prognostic relevance of MMP-11 expression in the stromal fibroblast-like cells adjacent to invasive ductal carcinoma of the breast. Annals of surgical oncology 20 Suppl 3: S433-442.
129. Kasper G, Reule M, Tschirschmann M, Dankert N, Stout-Weider K, et al. (2007) Stromelysin-3 over-expression enhances tumourigenesis in MCF-7 and MDA-MB-231 breast cancer cell lines: involvement of the IGF-1 signalling pathway. BMC cancer 7: 12.
130. Cheng CW, Yu JC, Wang HW, Huang CS, Shieh JC, et al. (2010) The clinical implications of MMP-11 and CK-20 expression in human breast cancer. Clinica chimica acta 411: 234-241.
131. Eiseler T, Doppler H, Yan IK, Goodison S, Storz P (2009) Protein kinase D1 regulates matrix metalloproteinase expression and inhibits breast cancer cell invasion. Breast cancer research 11: R13.
132. Garcia MF, Gonzalez-Reyes S, Gonzalez LO, Junquera S, Berdize N, et al. (2010) Comparative study of the expression of metalloproteases and their inhibitors in different localizations within primary tumours and in metastatic lymph nodes of breast cancer. International journal of experimental pathology 91: 324-334.
133. Eiro N, Fernandez-Garcia B, Gonzalez LO, Vizoso FJ (2013) Cytokines related to MMP-11 expression by inflammatory cells and breast cancer metastasis. Oncoimmunology 2: e24010.
134. Tan J, Buache E, Alpy F, Daguenet E, Tomasetto CL, et al. (2013) Stromal matrix metalloproteinase-11 is involved in the mammary gland postnatal development. Oncogene.
135. Selvey S, Haupt LM, Thompson EW, Matthaei KI, Irving MG, et al. (2004) Stimulation of MMP-11 (stromelysin-3) expression in mouse fibroblasts by cytokines, collagen and co-culture with human breast cancer cell lines. BMC cancer 4: 40.
136. Hegedus L, Cho H, Xie X, Eliceiri GL (2008) Additional MDA-MB-231 breast cancer cell matrix metalloproteinases promote invasiveness. Journal of cellular physiology 216: 480-485.
137. Min KW, Kim DH, Do SI, Pyo JS, Kim K, et al. (2012) Diagnostic and Prognostic Relevance of MMP-11 Expression in the Stromal Fibroblast-Like Cells Adjacent to Invasive Ductal Carcinoma of the Breast. Annals of surgical oncology 3: S433-442.
138. Ueki T, Park JH, Nishidate T, Kijima K, Hirata K, et al. (2009) Ubiquitination and downregulation of BRCA1 by ubiquitin-conjugating enzyme E2T overexpression in human breast cancer cells. Cancer Research 69: 8752-8760.
139. Ellsworth RE, Seebach J, Field LA, Heckman C, Kane J, et al. (2009) A gene expression signature that defines breast cancer metastases. Clinical & experimental metastasis 26: 205-213.
140. Kim H, Watkinson J, Varadan V, Anastassiou D (2010) Multi-cancer computational analysis reveals invasion-associated variant of desmoplastic reaction involving INHBA, THBS2 and COL11A1. BMC medical genomics 3: 51.
141. Li Y, Wu J, Zhang W, Zhang N, Guo H (2013) Identification of serum CCL15 in hepatocellular carcinoma. British journal of cancer 108: 99-106.
142. Itatani Y, Kawada K, Fujishita T, Kakizaki F, Hirai H, et al. (2013) Loss of SMAD4 from colorectal cancer cells promotes CCL15 expression to recruit CCR1+ myeloid cells and facilitate liver metastasis. Gastroenterology 145: 1064-1075.
143. van Zalen S, Nijenhuis M, Jonkman MF, Pas HH (2006) Two major 5'-untranslated regions for type XVII collagen mRNA. Journal of dermatological science 43: 11-19.

144. Wang J, He Q, Shao YG, Ji M (2013) Chemokines fluctuate in the progression of primary breast cancer. European Review for Medical and Pharmacological Sciences 17: 596-608.
145. Bandyopadhyay S, Zhan R, Chaudhuri A, Watabe M, Pai SK, et al. (2006) Interaction of KAI1 on tumor cells with DARC on vascular endothelium leads to metastasis suppression. Nature medicine 12: 933-938.
146. Zeng XH, Ou ZL, Yu KD, Feng LY, Yin WJ, et al. (2011) Coexpression of atypical chemokine binders (ACBs) in breast cancer predicts better outcomes. Breast cancer research and treatment 125: 715-727.
147. Liu XF, Li LF, Ou ZL, Shen R, Shao ZM (2012) Correlation between Duffy blood group phenotype and breast cancer incidence. BMC cancer 12: 374.
148. Wu J, Liu S, Liu G, Dombkowski A, Abrams J, et al. (2012) Identification and functional analysis of 9p24 amplified genes in human breast cancer. Oncogene 31: 333-341.
149. Bernardo GM, Keri RA (2012) FOXA1: a transcription factor with parallel functions in development and cancer. Bioscience reports 32: 113-130.
150. Ni M, Chen Y, Lim E, Wimberly H, Bailey ST, et al. (2011) Targeting androgen receptor in estrogen receptor-negative breast cancer. Cancer cell 20: 119-131.
151. Augello MA, Hickey TE, Knudsen KE (2011) FOXA1: master of steroid receptor function in cancer. The EMBO journal 30: 3885-3894.
152. Robinson JL, Macarthur S, Ross-Innes CS, Tilley WD, Neal DE, et al. (2011) Androgen receptor driven transcription in molecular apocrine breast cancer is mediated by FoxA1. The EMBO journal 30: 3019-3027.
153. Bernardo GM, Lozada KL, Miedler JD, Harburg G, Hewitt SC, et al. (2010) FOXA1 is an essential determinant of ERalpha expression and mammary ductal morphogenesis. Development 137: 2045-2054.
154. Robinson JL, Carroll JS (2012) FoxA1 is a key mediator of hormonal response in breast and prostate cancer. Frontiers in endocrinology 3: 68.
155. Fu X, Huang C, Schiff R (2011) More on FOX News: FOXA1 on the horizon of estrogen receptor function and endocrine response. Breast cancer research 13: 307.
156. Robinson JL, Holmes KA, Carroll JS (2013) FOXA1 mutations in hormone-dependent cancers. Frontiers in oncology 3: 20.
157. Meyer KB, Carroll JS (2012) FOXA1 and breast cancer risk. Nature genetics 44: 1176-1177.
158. Cowper-Sal lari R, Zhang X, Wright JB, Bailey SD, Cole MD, et al. (2012) Breast cancer risk-associated SNPs modulate the affinity of chromatin for FOXA1 and alter gene expression. Nature genetics 44: 1191-1198.
159. Katika MR, Hurtado A (2013) A functional link between FOXA1 and breast cancer SNPs. Breast Cancer Res 15: 303.
160. Badve S, Turbin D, Thorat MA, Morimiya A, Nielsen TO, et al. (2007) FOXA1 expression in breast cancer--correlation with luminal subtype A and survival. Clinical cancer research 13: 4415-4421.
161. Yamaguchi N, Ito E, Azuma S, Honma R, Yanagisawa Y, et al. (2008) FoxA1 as a lineage-specific oncogene in luminal type breast cancer. Biochemical and biophysical research communications 365: 711-717.
162. Mehta RJ, Jain RK, Leung S, Choo J, Nielsen T, et al. (2012) FOXA1 is an independent prognostic marker for ER-positive breast cancer. Breast cancer research and treatment 131: 881-890.
163. Habashy HO, Powe DG, Rakha EA, Ball G, Paish C, et al. (2008) Forkhead-box A1 (FOXA1) expression in breast cancer and its prognostic significance. European journal of cancer 44: 1541-1551.

164. Hisamatsu Y, Tokunaga E, Yamashita N, Akiyoshi S, Okada S, et al. (2012) Impact of FOXA1 expression on the prognosis of patients with hormone receptor-positive breast cancer. Annals of surgical oncology 19: 1145-1152.
165. Hisamatsu Y, Tokunaga E, Yamashita N, Akiyoshi S, Okada S, et al. (2014) Impact of GATA-3 and FOXA1 expression in patients with hormone receptor-positive/HER2-negative breast cancer. Breast cancer.
166. Albergaria A, Paredes J, Sousa B, Milanezi F, Carneiro V, et al. (2009) Expression of FOXA1 and GATA-3 in breast cancer: the prognostic significance in hormone receptor-negative tumours. Breast cancer research 11: R40.
167. Hurtado A, Holmes KA, Ross-Innes CS, Schmidt D, Carroll JS (2011) FOXA1 is a key determinant of estrogen receptor function and endocrine response. Nature genetics 43: 27-33.
168. Naderi A, Meyer M, Dowhan DH (2012) Cross-regulation between FOXA1 and ErbB2 signaling in estrogen receptor-negative breast cancer. Neoplasia 14: 283-296.
169. Kong SL, Li G, Loh SL, Sung WK, Liu ET (2011) Cellular reprogramming by the conjoint action of ERalpha, FOXA1, and GATA3 to a ligand-inducible growth state. Molecular systems biology 7: 526.
170. Magnani L, Lupien M (2014) Chromatin and epigenetic determinants of estrogen receptor alpha (ESR1) signaling. Molecular and cellular endocrinology 382: 633-641.
171. Lasa A, Garcia A, Alonso C, Millet P, Cornet M, et al. (2013) Molecular detection of peripheral blood breast cancer mRNA transcripts as a surrogate biomarker for circulating tumor cells. PloS one 8: e74079.
172. Lacroix M (2006) Significance, detection and markers of disseminated breast cancer cells. Endocrine-Related Cancer 13: 1033–1067.
173. Ahmed AR, Griffiths AB, Tilby MT, Westley BR, May FE (2012) TFF3 is a normal breast epithelial protein and is associated with differentiated phenotype in early breast cancer but predisposes to invasion and metastasis in advanced disease. The American journal of pathology 180: 904-916.
174. Fenne IS, Helland T, Flageng MH, Dankel SN, Mellgren G, et al. (2013) Downregulation of steroid receptor coactivator-2 modulates estrogen-responsive genes and stimulates proliferation of mcf-7 breast cancer cells. PloS one 8: e70096.
175. Roll JD, Rivenbark AG, Sandhu R, Parker JS, Jones WD, et al. (2013) Dysregulation of the epigenome in triple-negative breast cancers: basal-like and claudin-low breast cancers express aberrant DNA hypermethylation. Experimental and molecular pathology 95: 276-287.
176. Sandhu R, Rivenbark AG, Mackler RM, Livasy CA, Coleman WB (2014) Dysregulation of microRNA expression drives aberrant DNA hypermethylation in basal-like breast cancer. International journal of oncology 44: 563-572.
177. Wang J, Ray PS, Sim MS, Zhou XZ, Lu KP, et al. (2012) FOXC1 regulates the functions of human basal-like breast cancer cells by activating NF-kappaB signaling. Oncogene 31: 4798-4802.
178. Sizemore ST, Keri RA (2012) The forkhead box transcription factor FOXC1 promotes breast cancer invasion by inducing matrix metalloprotease 7 (MMP7) expression. The Journal of biological chemistry 287: 24631-24640.
179. Tkocz D, Crawford NT, Buckley NE, Berry FB, Kennedy RD, et al. (2012) BRCA1 and GATA3 corepress FOXC1 to inhibit the pathogenesis of basal-like breast cancers. Oncogene 31: 3667-3678.

180. Andres SA, Brock GN, Wittliff JL (2013) Interrogating differences in expression of targeted gene sets to predict breast cancer outcome. BMC Cancer 13: 1-18.
181. Symmans WF, Fiterman DJ, Anderson SK, Ayers M, Rouzier R, et al. (2005) A single-gene biomarker identifies breast cancers associated with immature cell type and short duration of prior breastfeeding. Endocrine-related cancer 12: 1059-1069.
182. Bell JL, Wachter K, Muhleck B, Pazaitis N, Kohn M, et al. (2013) Insulin-like growth factor 2 mRNA-binding proteins (IGF2BPs): post-transcriptional drivers of cancer progression? Cellular and molecular life sciences 70: 2657-2675.
183. Fadare O, Liang SX, Crispens MA, Jones HW, 3rd, Khabele D, et al. (2013) Expression of the oncofetal protein IGF2BP3 in endometrial clear cell carcinoma: assessment of frequency and significance. Human pathology 44: 1508-1515.
184. Lochhead P, Imamura Y, Morikawa T, Kuchiba A, Yamauchi M, et al. (2012) Insulin-like growth factor 2 messenger RNA binding protein 3 (IGF2BP3) is a marker of unfavourable prognosis in colorectal cancer. European journal of cancer 48: 3405-3413.
185. Samanta S, Pursell B, Mercurio AM (2013) IMP3 protein promotes chemoresistance in breast cancer cells by regulating breast cancer resistance protein (ABCG2) expression. The Journal of biological chemistry 288: 12569-12573.
186. Samanta S, Sharma VM, Khan A, Mercurio AM (2012) Regulation of IMP3 by EGFR signaling and repression by ERbeta: implications for triple-negative breast cancer. Oncogene 31: 4689-4697.
187. Won JR, Gao D, Chow C, Cheng J, Lau SY, et al. (2013) A survey of immunohistochemical biomarkers for basal-like breast cancer against a gene expression profile gold standard. Modern pathology 26: 1438-1450.
188. Walter O, Prasad M, Lu S, Quinlan RM, Edmiston KL, et al. (2009) IMP3 is a novel biomarker for triple negative invasive mammary carcinoma associated with a more aggressive phenotype. Human pathology 40: 1528-1533.
189. Bu D, Lewis CM, Sarode V, Chen M, Ma X, et al. (2013) Identification of Breast Cancer DNA Methylation Markers Optimized for Fine-Needle Aspiration Samples. Cancer epidemiology, biomarkers & prevention 22: 2212-2221.
190. Martens JW, Nimmrich I, Koenig T, Look MP, Harbeck N, et al. (2005) Association of DNA methylation of phosphoserine aminotransferase with response to endocrine therapy in patients with recurrent breast cancer. Cancer research 65: 4101-4117.
191. Lan J, Zhao J, Liu Y (2012) Molecular cloning, sequence characterization, polymorphism and association analysis of porcine ROPN1 gene. Molecular biology reports 39: 2739-2743.
192. Ivanov SV, Panaccione A, Nonaka D, Prasad ML, Boyd KL, et al. (2013) Diagnostic SOX10 gene signatures in salivary adenoid cystic and breast basal-like carcinomas. British journal of cancer 109: 444-451.
